# Cystatin B increases autophagic flux by sustaining proteolytic activity of cathepsin B and fuels glycolysis in pancreatic cancer: CSTB orchestrates autophagy and glycolysis in PDAC

**DOI:** 10.1002/ctm2.1126

**Published:** 2022-12-10

**Authors:** Yongsheng Jiang, Lijie Han, Meilin Xue, Ting Wang, Youwei Zhu, Cheng Xiong, Minmin Shi, Hongzhe Li, Wangxi Hai, Yanmiao Huo, Baiyong Shen, Lingxi Jiang, Hao Chen

**Affiliations:** ^1^ Department of General Surgery Pancreatic Disease Center Ruijin Hospital Shanghai Jiao Tong University School of Medicine Shanghai China; ^2^ Research Institute of Pancreatic Diseases Shanghai Jiao Tong University School of Medicine Shanghai China; ^3^ State Key Laboratory of Oncogenes and Related Genes Shanghai Jiao Tong University School of Medicine Shanghai China; ^4^ Department of Pathology Ruijin Hospital Shanghai Jiao Tong University School of Medicine Shanghai China; ^5^ Department of Nuclear Medicine Ruijin Hospital Shanghai Jiao Tong University School of Medicine Shanghai China; ^6^ Department of Biliary‐Pancreatic Surgery Renji Hospital School of Medicine Shanghai Jiao Tong University Shanghai P. R. China; ^7^ Institute of Translational Medicine Shanghai Jiao Tong University Shanghai China

**Keywords:** autophagy, biomarker, cystatin B, cystatin C, cathepsin B, glycolysis, pancreatic ductal adenocarcinoma

## Abstract

**Background:**

Both autophagy and glycolysis are essential for pancreatic ductal adenocarcinoma (PDAC) survival due to desmoplasia. We investigated whether targeting a hub gene which participates in both processes could be an efficient strategy for PDAC treatment.

**Methods:**

The expression pattern of glycolysis signatures (GS) and autophagy signatures (AS) and their correlation with cystatin B (CSTB) in PDAC were analysed. It was discovered how CSTB affected the growth, glycolysis, and autophagy of PDAC cells. We assessed competitive binding to cathepsin B (CTSB) between CSTB and cystatin C (CSTC) via immunoprecipitation (IP) and immunofluorescence (IF). Chromatin immunoprecipitation quantitative polymerase chain reaction (ChIP‐qPCR) and luciferase reporter gene assays were used to unveil the mechanism underlying CSTB upregulation. The expression pattern of CSTB was examined in clinical samples and KrasG12D/+, Trp53R172H/+, Pdx1‐Cre (KPC) mice.

**Results:**

GS and AS were enriched and closely associated in PDAC tissues. CSTB increased autophagic flux and provided substrates for glycolysis. CSTB knockdown attenuated the proliferation of PDAC cells and patient‐derived xenografts. The liquid chromatography‐tandem mass spectrometry assay indicated CSTB interacted with CTSB and contributed to the proteolytic activity of CTSB in lysosomes. IF and IP assays demonstrated that CSTB competed with CSTC to bind to CTSB. Mutation of the key sites of CSTB abolished the interaction between CSTB and CTSB. CSTB was highly expressed in PDAC due to H3K27acetylation and SP1 expression. High expression of CSTB in PDAC was observed in tissue microarray and patients’ serum samples.

**Conclusions:**

Our work demonstrated the tumorigenic roles of autophagy and glycolysis in PDAC. CSTB is a key role in orchestrating these processes to ensure energy supply of PDAC cells.

## Graphical abstract

- Both glycolysis and autophagy processes were obviously active in pancreatic ductal adenocarcinoma (PDAC).
- Cystatin B (CSTB) promotes autophagic flux by competing with cystatin C to bind to cathepsin B and provides substrates for glycolysis in PDAC.
- High H3K27ac levels in the promoter region and SP1 upregulation contributed to the high expression of CSTB.

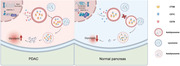

## 1 BACKGROUND

Pancreatic ductal adenocarcinoma (PDAC) is expected to rank as the second greatest cause of cancer deaths in the next 20 to 30 years, despite improvements in tumour identification and therapy.^1^ Macroautophagy (hereafter referred to as ‘autophagy’) is a biological process in which cellular material is transported to lysosomes for breakdown, resulting in the basal turnover of cell components and supplying macromolecular precursors. Autophagy generates diverse metabolic fuel sources to meet the increased anabolic needs of cancer cells and can allow them to thrive in hostile environments.^2^ For example, autophagy is able to activate amino acid biosynthetic pathways that serve as substrates for adenosine triphosphate (ATP) production or de novo protein synthesis.^3^ Advances in the understanding of autophagy in cancer have come a long way, indicating an opposing and context‐dependent role of autophagy in tumorigenesis.^4^ On the one hand, mice heterozygous for *Beclin 1*, whose autophagic activity is impaired, are prone to hepatocellular carcinoma.^5^ On the other hand, cancer cells with Ras mutations rely heavily on autophagy. PDAC, 95% of which harbour *KRAS* mutations, is characterised by autophagy‐dependent energy supplement and tumorigenic growth.^6^ Genetic or pharmacologic inhibition of autophagy lead to impaired metabolism in PDAC.^2^, ^7^, ^8^ Although autophagy is critical for PDAC progression, how autophagy fuel cellular metabolism in PDAC is still obscure.

Tumour cells prefer glycolysis to the more energy‐efficient oxidative phosphorylation when metabolising glucose, even in the presence of oxygen, described as Warburg effect.^9^, ^10^ Glycolysis is able to divert glucose into side branches that produce key metabolites such as nicotinamide adenine dinucleotide phosphate (NADPH) and ribose 5‐phosphate to provide cells with metabolic plasticity.^11^, ^12^ As one of the most hypoxic human tumours, PDAC strongly tends to generate energy through glycolysis.^13^ The glucose supply of glycolysis can be largely affected by autophagy, which degrades carbohydrates into sugars and DNA into nucleosides to provide tumour cells with sufficient glycolytic substrates.^5^ Both glycolysis and autophagy are indispensable for PDAC to survive in the setting of a hostile tumor microenvironment (TME). However, the mechanism of inter‐regulatory network of autophagy and glycolysis remains obscure.

In this study, function‐based gene signatures were established to investigate molecular mechanism shared in glycolysis and autophagy. Cystatin B (CSTB) was identified as a hub gene. CSTB, a cysteine protease (cathepsin) inhibitor of the cystatin superfamily, functions to limit the excessive activity of cysteine proteases by forming tight complexes.^14^, ^15^ Given the primary role of cathepsin in lysosome proteolysis, imbalance between CSTB and cathepsin often leads to impaired autophagy and is associated with multiple diseases including cancer.^16^, ^17^ The role of CSTB in different types of cancers is controversial. CSTB promotes tumour growth in PyMT murine model.^18^ Upregulation of CSTB has also been observed in hepatocellular carcinoma and ovarian clear cell carcinoma.^19^, ^20^ Differently, knockdown of CSTB in gastric cancer cells lead to impaired proliferation and migration.^21^ However, the role of CSTB in PDAC is still limited. Molecular characterisation of CSTB may provide an avenue for developing glycolysis‐ and autophagy‐targeting therapeutic intervention strategies for PDAC treatment.

Here, we revealed that both glycolysis and autophagy were hyperactive and closely correlated with each other in PDAC. CSTB increases autophagic flux by competing with cystatin C (CSTC) to bind to cathepsin B (CTSB) and sustaining the proteolytic activity of CTSB. Thereafter, increased autophagic flux facilitates the supply of substrates for biosynthesis and fuels glycolysis in PDAC. High levels of CSTB expression are linked to a poor prognosis and may be utilized as a serum marker in PDAC.

## 2 METHODS

### 2.1 Data mining and bioinformatics analysis

The Gene Expression Omnibus (GEO), The Cancer Genome Atlas (TCGA), and Genotype‐Tissue Expression (GTEx) databases were used to gather and download gene expression data with associated clinical data of patients and healthy individuals. Gene expression profiles for the TCGA and GTEx datasets were obtained from the data hubs at UCSC Xena (https://xenabrowser.net/hub/). The robust multi‐array averaging technique was used to standardise the microarray data. We selected a 7‐gene glycolysis signature (GS) and a 68‐gene autophagy signature (AS) that have shown great performance in classifying patients with different overall survival rates. These gene signatures were identified using data on gene function, in vivo co‐expression analyses, and relevant literature. To test the robustness of the defined signatures, we collected eight glycolysis or autophagy‐related GEO datasets from multiple cancer types. Glycolysis and autophagy scores were calculated via gene set variation analysis (GSVA) based on the expression of GS and AS in the corresponding datasets. GSVA is a specific sort of gene set enhancement strategy that deals with single samples and empowers pathway‐driven examinations of sub‐atomic information by playing out a thoughtfully straightforward yet strong change in the practical unit of analysis, from genes to gene sets.^22^ High GS/AS Group and Low GS/AS Group were determined based on the GSVA score. Samples with top 50% GSVA score were defined as high group, otherwise they were classified as low group. Samples with 2DG, high SUVmax, high stemness, and hypoxia were defined as high glycolysis, whereas samples with OE‐TFEB, starvation, and ATO were defined as high autophagy. To decide the connection between the characterised signature and in vivo organic ways of behaving, using the ‘GSVA’ R package, we assessed the single‐sample gene set enrichment analysis results obtained when comparing the gene profiles of tests from the TCGA, GTEx, and GEO. An enrichment score compared to every mark was acquired for each example, and the relationship between these marks and in vivo organic ways of behaving was determined. Differential articulation quality among PDAC and neighbouring tissue were investigated utilising the ‘limma’ bundle in R.

### 2.2 Cell culture

Every cell line was grown at 37°C and 5% CO_2_. Dulbecco's modified Eagle medium (DMEM) containing 10% fetal bovine serum (FBS) and 50 μg/ml penicillin/streptomycin (P/S) were used to culture Patu 8988, and Panc1 cells. Iscove's modified Eagle medium (IMDM) containing 10% FBS and 50 μg/ml penicillin/streptomycin (P/S) were used to culture CFPAC‐1 cells. All cells were checked routinely for the absence of mycoplasma contamination. Short tandem repeat profiling is used for authentication of all cell lines.

### 2.3 Knockdown and overexpression assays

Two short hairpin RNAs specifically targeting CSTB (sh‐CSTB‐1 and sh‐CSTB‐2), CSTB‐overexpressing (OE‐CSTB), and CSTB‐mutant cell lines were generated using lentiviral vectors. For virus creation, Lipofectamine 2000 (Invitrogen, USA) with 4 μg of each plasmid and 4 μg of helper plasmid (2 μg pMD2G and 2 μg of psPAX2) were transfected into 293T cells. Then, 48 h later, a 0.45 m filter was used to filter the viral supernatants. The 300 000 PDAC cells were combined and infected for 6 h with viral supernatants and 10 g/ml polybrene (Yeasen). To create stable cell lines, transfected cells were chosen using puromycin at a concentration of 10 g/ml. Lipofectamine 2 000 was selected to deliver siRNAs, as indicated by the producer's guidelines. Data of plasmids and siRNA is depicted in the Extended Information.

### 2.4 Quantitative real‐time PCR

HiScript^®^ Reverse Transcriptase (Vazyme, China) was used to conduct reverse transcription after total RNA was extracted using the TRIzol Reagent as indicated by the producer's guidelines. For quantitative PCR analysis, a SYBR Green PCR Kit (Vazyme, China) was used to amplified double‐stranded cDNA and detected using qTOWER384G (Analytikjena). A list of the primer sequences utilised in this investigation is contained in the Extended Information.

### 2.5 Immunoblotting and immunoprecipitation

To extract cellular protein, we lyse cell samples with ice‐cold RIPA buffer (Solarbio; China) pre‐mixed with protease and phosphatase inhibitor cocktail (Biotech, China). Cells were mechanically ruptured by a Cell Lifter (Corning) at least 10 times. Lysates were centrifuged at 12 000 rpm for 15 min, measured with bicinchoninic acid (BCA), diluted in Marker Sample Buffer (Thermo Scientific), and boiled for 15 min. To perform immunoblotting, proteins were isolated by sodium dodecyl sulfate polyacrylamide gel electrophoresis (SDS‐PAGE) and moved onto polyvinylidene fluoride (PVDF) membranes. The 5% bovine serum albumin (Proliant, New Zealand) in Tris‐buffered saline with 0.1% Tween 20 (Sangon Biotech) was used to block the membranes. After that, the membranes were treated with the appropriate primary and secondary antibodies. The Extended Information includes a list of the antibodies utilised.

For immunoprecipitation, a mild lysis buffer (Beyotime) was used to lyse cells. Cell lysates were centrifuged for 15 min at 4°C and supernatants were incubated with protein G agarose (Beyotime) for 1 h at 4°C. Then we collected supernatant and subjected it to immunoprecipitation. The antibody was used to incubate the supernatant at 4°C with gentle rocking overnight. Cell lysis buffer was used to wash the pellets four times after the samples were centrifuged at 4°C. Before performing an immunoblot, the pellets were reconstituted in a Marker Sample Buffer from Thermo Scientific and heated for 15 min. Information on antibodies is shown in the Extended Information.

### 2.6 Seahorse analyses

The assay was performed as indicated by the producer's guidelines. In a nutshell, CFPAC and Patu8988 cells were cultivated in a XF 96‐well plate at a thickness of 1×10^4^ cells per well. To determine the effect of bafilomycin A1 (BAF), cells were incubated overnight at 37°C with the specified BAF concentration. The cells were rinsed with phosphate‐buffered saline (PBS) upon the next day, and the culture medium was replaced with test media 1 h before the measurement. For the glycolytic stress test, each well was added with 10 mmol/L glucose, 2 μmol/L oligomycin, and 50 mmol/L 2‐deoxyglucose (2‐DG). For the mitochondrial stress test, each well was added with 1 μmol/L oligomycin, 2 μmol/L FCCP, 0.5 μmol/L rotenone, and 0.5 mmol/L actinomycin A. The two estimations were standardised to the all out protein quantitation.

### 2.7 Glucose, ATP, and lactate measurement

To perform glycose and lactate measurements, we seeded cells in a 24‐well plate culture dishes, followed by treatment with the corresponding reagents 1 day later. The supernatants were collected by centrifugation, and then used to measure glucose and lactate concentrations. Glucose consumption was calculated as indicated by the producer's guidelines. ATP and lactate production was measured using the ATP detection kit (Beyotime) and Lactate Assay Kit (Abcam, ab65331).

### 2.8 PDAC transgenic model, subcutaneous xenograft model, orthotopic xenograft model, and patient‐derived xenograft model

This study's PDAC transgenic mouse model was generated by crossing Pdx1‐Cre mice with lox‐stop‐lox‐Kras^G12D/+^ and lox‐stop‐loxTrp53^R172H/+^ (KPC). KPC mouse pancreas tissues were obtained at 18‐20 weeks. Athymic male nu/nu mice (BALB/c) aged 6‐8 weeks used in this study were purchased from the Chinese Academy of Sciences (Shanghai, China). To build subcutaneous implant models, 5×106 cells in 100 μl PBS were subcutaneous injected. The mice were sacrificed after 30 days. The xenografts were then isolated and gauged. Orthotopic xenograft models and patient‐derived xenograft (PDX) models were utilised, as reported previously.^23^, ^24^ The PDX model were generated using three individual PDAC tissues. For adenovirus‐associated virus (AAV) injection, F3 PDX models were used in investigations. When the volume of the tumour reached 50 mm3 (8 weeks after transplantation), AAV was administered intratumoural twice each week for 4 weeks. After 4 weeks, the mice were sacrificed, the tumour was separated, and its weight was determined. At four distinct tumour body injection locations, 1^10^ AAV particles dispersed in 20 μl PBS were injected into PDX animals. AAVs carrying the shRNA‐targeting CSTB and NC shRNA were packaged by Bioegene (Shanghai, China) using the AAV2/9 serotype. The targeting sequences are as follows: shCSTB: CTGTGTTTAAGGCCGTGTCAT and NC: CCTAAGGTTAAGTCGC‐CCTCG. Mice was sacrificed 4 weeks after treatment.

For 18F‐fluoro‐D‐glucose (FDG) PET‐CT experiments, mice were sedated with chloral hydrate and given 7.4 MBq of 18F‐FDG intravenously. Using an Inveon Animal‐PET/CT scanner (Siemens Preclinical Solution, Knoxville, TN), PET and CT images were collected after a 1 h uptake period. The PET‐CT images were combined using the Inveon Research Workplace (Siemens Preclinical Solution, Knoxville, TN) and three‐dimensional ordered‐subset expectation maximisation (OSEM3D)/maximum algorithms. Using the maximal standardised uptake value (SUVMax) inside the tumour, a qualitative evaluation of the changes in FDG uptake was done.

The mice were housed in a microbe free research centre creature unit at Ruijin Hospital affiliated with Shanghai Jiao Tong University School of Medicine. All animal tests were authorised by the hospital's ethics committee

### 2.9 Immunohistochemistry and immunofluorescence

The Ethic Committees of Ruijin Hospital reviewed and examined and authorised studies involving human tissues. Protein expression in PDAC tissues was determined using immunohistochemistry (IHC) in formalin‐fixed paraffin‐embedded (FFPE) tumours from Ruijin Hospital surgery patients. Tumour tissues were assessed according to the percentage of stained area (0 = 0%‐5%, 1 = 6%‐35%, 2 = 36%‐70%, 3 = more than 70%) and the degree of the staining of the nuclei or cytoplasm (0 = no staining, 1 = weak staining, 2 = moderate staining, and 3 = strong staining). Final scores were calculated by multiplying the two numbers listed above (“negative/−” for scores 0‐1; “weak/+” for scores 2‐3; “moderate/++” for scores 4‐6; and “strong/+++” for scores more than 6). Those with scores over 4 were deemed to have high expression, whereas samples with scores below 4 were deemed to have low expression. In the Extended Information, specific antibodies used for IHC are outlined.

To assess LC3, P62, CTSB, and CSTC distribution, CFPAC and Patu 8988 cells were seeded on no. 1.5 cover glass (2850‐22, Corning). Immunofluorescence (IF) experiments were carried out in accordance with standard protocols. Cells were fixed for 15 min with 4% paraformaldehyde (Servicebio), washed, and permeabilised with 0.5% Triton‐X100 (Sigma) for 5 min. The cells were rinsed with PBS, blocked with 3% BSA containing 0.05% Tween 20, and 0.08% sodium azide for 1 h, then incubated with primary antibodies diluted in blocking solution for 1 h at 37°C. The cells were incubated with goat‐anti‐rabbit antibody for 30 min at room temperature after three 5 min washes in PBS. Cells were washed three times for 5 min in PBS, with the first wash containing 0.1 μg/ml DAPI (Thermo Scientific) or TUNEL (G1501, Servicebio) for TUNEL detection assay and mounted with Vectashield antifade mounting medium (H‐1 000, Vector Labs) for microscopy.

To quantify the ratio of autophagosomes to autolysosomes, we first counted the number of autophagosomes (red dots count) and autolysosomes (yellow dots count), respectively. After a tandem mCherry‐enhanced green fluorescent protein‐LC3B expression plasmid transfected into cells, the GFP fluorescence will quench in an acidic lysosomal environment if autophagic flux was not obstructed. In that case, red and only both expressing dots (merged as yellow dots) represents autophagosomes for they were not fused with lysosomes.

### 2.10 Transmission electron microscopy

CFPAC or Patu 8988 cells were initially fixed with freshly made 2.5% glutaraldehyde (Servicebio) at 4°C and then fixed with 1% OsO4 buffer (pH 7.2) for at least 2 h at 4°C. The cells were washed with buffer solution, dehydrated in acetone with different gradient, embedded with electron microscopic embedding media, localised with semithin section and carefully sliced into 60‐nm sections for further staining. Uranyl acetate and lead citrate were utilised for staining of ultrathin sections before being examined with a transmission electron microscope (HT7800, Hitachi).

### 2.11 Cell proliferation assays

The Cell Counting Kit‐8 (CCK‐8) was used to calculate the cell proliferation rate in accordance with the manufacturer's protocol (Meilunbio, China). 3×103 CFPAC or Patu 8988 cells were seeded in 96‐well plates supplemented with 100 μl complete medium with 10 % FBS to evaluate the cell proliferation rate. The value at 450 nm wavelength was measured (Biotek, USA) every 24 h for at least 6 days. 1×103 CFPAC and Patu 8988 cells were seeded in 6‐well plates for 10 days for colony formation assays, followed by staining with crystal violet (Beyotime) dye for 20 min. An EdU kit (BeyoClick^™^ EdU Cell Proliferation Kit with Alexa Fluor 555, Beyotime, China) was utilised to assess EdU proliferation. In brief, 1 × 106 CFPAC or Patu 8988 cells were seeded in 6‐well plates. After incubating with EdU solution for 3 h, cells were fixed with 4% paraformaldehyde at room temperature for 15  min, and permeabilised with 0.3% Triton X‐100 for 15  min. The cells were incubated with the click reaction mixture at room temperature for 30  min in the dark, followed by 15  min incubation with Hoechst 33342.

### 2.12 Liquid chromatography‐mass spectrometry

The liquid chromatography‐mass spectrometry (LC‐MS) was utilised on CO‐IP samples to identify the potential target of cathepsin. Briefly, cell lysates were immunoprecipitated with anti‐CSTB and protein A/G beads. SDS‐PAGE was used to separate the proteins, and bands corresponding to the CSTB gene were removed, reduced, alkylated, and trypsinised. Thermo Fisher Scientific's Q Exactive HF‐mass spectrometer in conjunction with Proxeon Biosystems' Easy nLC were used to examine tryptic peptides for 60 min.

The mass spectrometer was set in positive ion mode. The data‐dependent top 10 method dynamically choosing the most prevalent presursor ions from the survey scan (300‐1800 *m*/*z*) for high‐energy collision dissociation (HCD) fragmentation was used for gathering MS data. The automatic gain control target was 3^6^, and maximum inject time was set to 10 ms. The duration of dynamic exclusion was set to 40.0 s. Survey scans were acquired at a resolution of 70 000 at *m*/*z* 200 and HCD spectra was acquired at a resolution of 17 500 at *m*/*z* 200, with an isolation width of 2 *m*/*z*. The normalised collision energy was 30 eV with an underfill ratio of 0.1%. The peptide recognition mode was enabled on the instrument.

MASCOT engine (version 2.2) was utilised to search MS/MS spectra against a nonredundant International Protein Index arabidopsis sequence database v3.85 from the European Bioinformatics Institute. The following initial options were utilised for protein identification. Peptide mass tolerance is 20 ppm, enzyme is trypsinised, MS/MS tolerance is 0.1 Da and missed cleavage is 2. Carbamidomethyl (C) is a fixed modification, whereas oxidation(M) is a variable modification.

The dataset identification for the mass spectrometry proteomics data is PXD037970, and it has been submitted to the ProteomeXchange Consortium (http://proteomecentral.proteomexchange.org) via the iProX partner repository.

### 2.13 Enzyme activity measurement

In vitro CTSB, CTSH, CTSL, and CTSC enzyme activity assays were performed using the Cathepsin Activity Kit (K140‐100,K145‐100, K142‐100; Biovision) (GenMed Scientific Inc., USA). CFPAC and Patu 8988 cell lysates were prepared, and a fluorescence plate reader (Biotek) was utilised to quantify the released fluorescence.

### 2.14 Construction of docking mode

Proteins with full‐length wild‐type sequences (CSTB Uniprot ID: P04080 and CTSB Uniprot ID: P07858) were collected from UniProt (https://www.uniprot.org/).^25^ The crystal structures of the proteins (CSTB PDB ID: 4N6V and CTSB PDB ID: 1HUC) were retrieved from the RCSB PDB (https://www.rcsb.org/).^26^ Before docking, water molecules, heteroatoms, and repeated subunits were removed using PyMOL. SwissModel (https://swissmodel.expasy.org/)^27^ was used for structural modelling of mutated CSTB G50E using PDB ID: 4N6V as a template, and the quality of the model was checked using PROCHECK (Ramachandran plot) and Verify 3D, using SAVES6.0.^28^ Zdock server 3.0.2 (https://zdock.umassmed.edu/) was used to dock the proteins CTSB and CSTB.^29^ The best docked complex of CTSB/CSTB docking using the ZDOCK server was selected for further hydrogen bond evaluation.

### 2.15 Chromatin immunoprecipitation

Chromatin immunoprecipitation (ChIP) analysis was carried out as previously descirbed.^30^ Briefly, the cells were quenched with glycine after being cross‐linked with 1% formaldehyde at room temperature for 10  min. After being sonicated to produce appropriate DNA fragments, the lysates were incubated with the H3K27ac antibodies. A negative control was employed, which was regular rabbit IgG. The primers of ChIP are listed in Extended Information.

### 2.16 Dual‐luciferase reporter assays

The pGL3 luciferase reporter vector was generated with a sequence including the potential SP1 binding site and the corresponding mutant region (Promega, Madison, WI, USA). These plasmids were introduced into 293T cells by transfection. A multi‐mode reader was used to measure the activity of luciferase (Biotek).

### 2.17 Quantification of CSTB levels in serum via ELISA

Following the manufacturer's instructions, blood plasma from healthy individuals and patients with PDAC was used to measure the levels of CSTB using a commercial ELISA kit (BPE10684, Lengton). Then, 5 ml of venous blood were drawn into an EDTA plasma tube. To avoid alternation of metabolites, the blood was immediately centrifuged at 1000 × *g* for 30 min at 4°C after collection to create platelet‐poor plasma samples. Plasma was carefully collected and kept at −80°C prior to measurement.

### 2.18 Statistical analysis

GraphPad Prism version 8.0 were selected to perform most statistical examinations. SPSS v23 were used to perform cox regression analyses as well as χ^2^ tests to investigate the correlation between clinical indicator and overall survival. Independent Student's *t*‐test was selected in two group comparation, and one‐way analysis of variance was utilised when comparing means between three or more subgroups. All cellular and molecular assays are replicate three times. The number of subcutaneous xenograft model and orthotopic xenograft model are five samples in each group. All statistical tests were two‐tailed, *p* < .05 was recognised as significance difference.

## 3 RESULTS

## 4 Glycolysis and autophagy are hyperactive in PDAC

**FIGURE 1:**
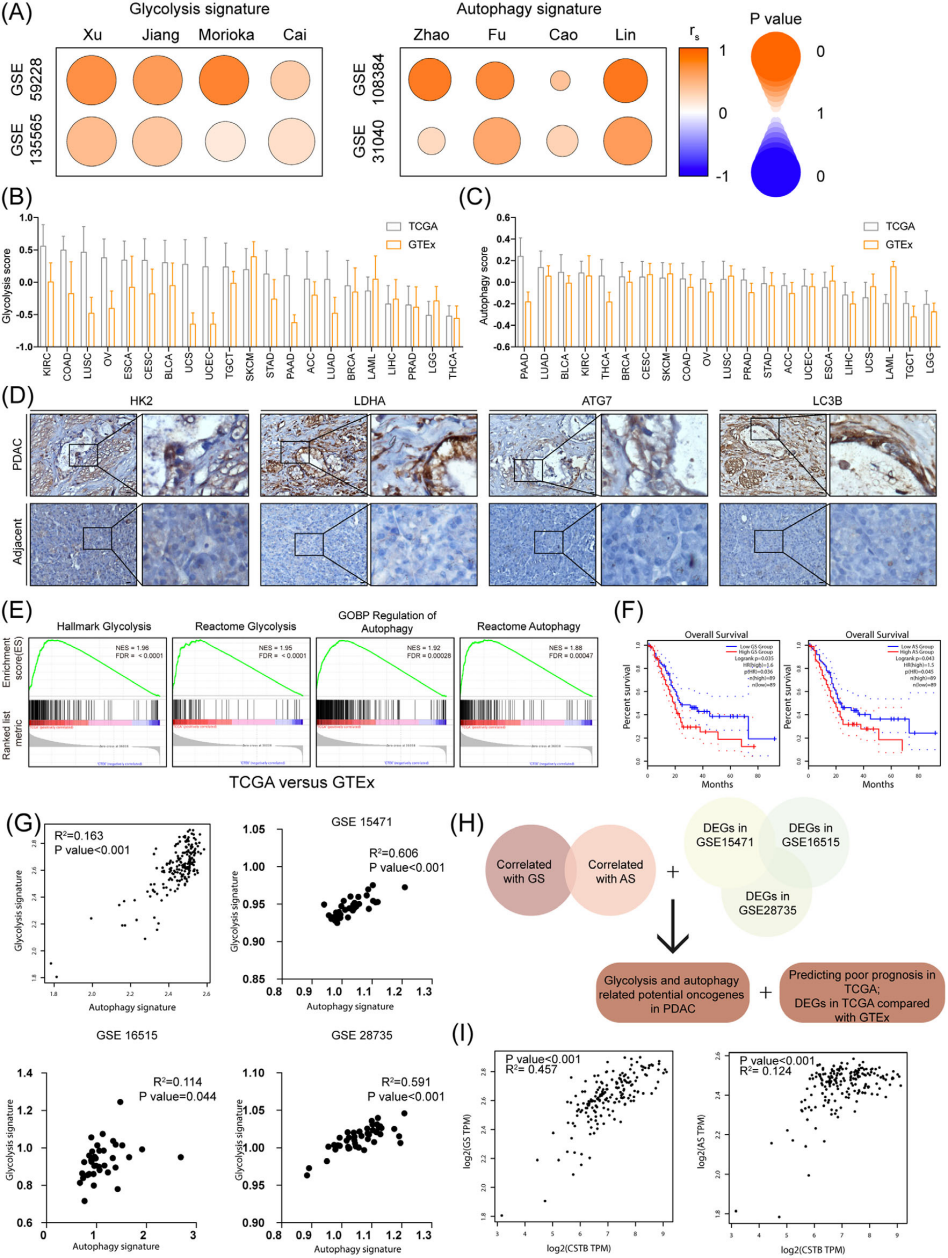
Evaluation of glycolysis and autophagy in pancreatic ductal adenocarcinoma (PDAC). (A) Spearman's correlation of Ruijin signature and the other eight gene signatures in glycolysis and autophagy‐related signatures, respectively. The colour intensity indicates Spearman's rank correlation coefficient (rs); the round size indicates *p* value for the Spearman's rank correlation. (B) Glycolysis score of TCGA tumour samples and corresponding GTEx normal tissue across cancer types. (C) Autophagy score of TCGA tumour samples and corresponding GTEx normal tissue across cancer types. (D) IHC images of HK2, LDHA, ATG7, and LC3B in PDAC samples and adjacent pancreas. (E) Gene set enrichment analysis plot based on the gene expression profiles between pancreatic cancer in TCGA and normal pancreas in GTEx. (F) Kaplan–Meier curves show that patients with high AS score were accompanied with worse overall survival (OS). (G) Analysis of correlation between glycolysis score and autophagy score in TCGA, GSE 15471, GSE16515, and GSE28735. (H) Diagram showing glycolysis and autophagy‐related gene screening strategy. (I) Correlation between CSTB and GS and AS respectively in TCGA datasets. Scale bar: 25 μm. *p < .05, **p < .01, ***p < .001, and ****p < .0001

To assess the roles of autophagy and glycolysis in pancreatic cancer, knowledge‐based functional gene expression signatures related to the two biological processes were selected and named as glycolysis signature (GS) and autophagy signature (AS), respectively (Table S1). By analysing eight glycolysis or autophagy‐related GEO datasets, we found that high‐glycolysis or high‐autophagy samples exhibited higher GS or AS score, respectively (Figure S1A,B). Moreover, GS and AS were positively correlated with other previous published signatures (Figure 1A).^31^, ^32^, ^33^, ^34^, ^35^, ^36^, ^37^, ^38^ Thus, it was reliable to use GS and AS to assess the levels of glycolysis and autophagy in different cancer types.

We selected 21 TCGA cancer types with corresponding normal tissue gene expression data in the GTEx for pan‐cancer analysis. The levels of glycolysis and autophagy in each sample of a certain cancer type was evaluated using GSVA based on GS and AS. Glycolysis is hyperactive in most cancer types, whereas the activity of autophagy varies (Figure 1B,C and Figure S1C). Compared to normal tissues, both of these biological processes were obviously active in PDAC from TCGA as well as three GEO datasets (Figure S1D). IHC staining of glycolytic and autophagic markers further confirmed the phenomenon in clinical PDAC samples (Figure 1D). As expected, gene set enrichment analysis (GSEA) indicated that autophagy and glycolysis‐related genes were enriched in PDAC compared to adjacent tissues as well (Figure 1E and Figure S1E). As shown in Figure 1F and Figure S1F, higher GS or AS scores were associated with poorer overall survival and disease‐free survival in PDAC clinical samples. Both of them were closely correlated with each other in TCGA and three GEO datasets, suggesting that an intra‐regulatory network of autophagy and glycolysis existed in PDAC (Figure 1G).

To identify the potential oncogenes participating in both glycolysis and autophagy, bioinformatics analysis and gene ontology analysis revealed a cluster of genes (LAMB3, CSTB, LAMC2, S100A6, ASAP2, PITX1, ZDHHC7, STIL, and HOXB5) that were closely associated with glycolysis and autophagy (Figure 1H). Among them, CSTB was the most significant differentially expressed gene in TCGA compared with GTEx and was associated with poorer prognosis (Figure 1H and Figure S1G–I). In line with this, CSTB exhibited a significantly positive correlation with AS and GS in TCGA (Figure 1I).

## 5 CSTB enhances autophagy flux and glycolysis in PDAC

**FIGURE 2:**
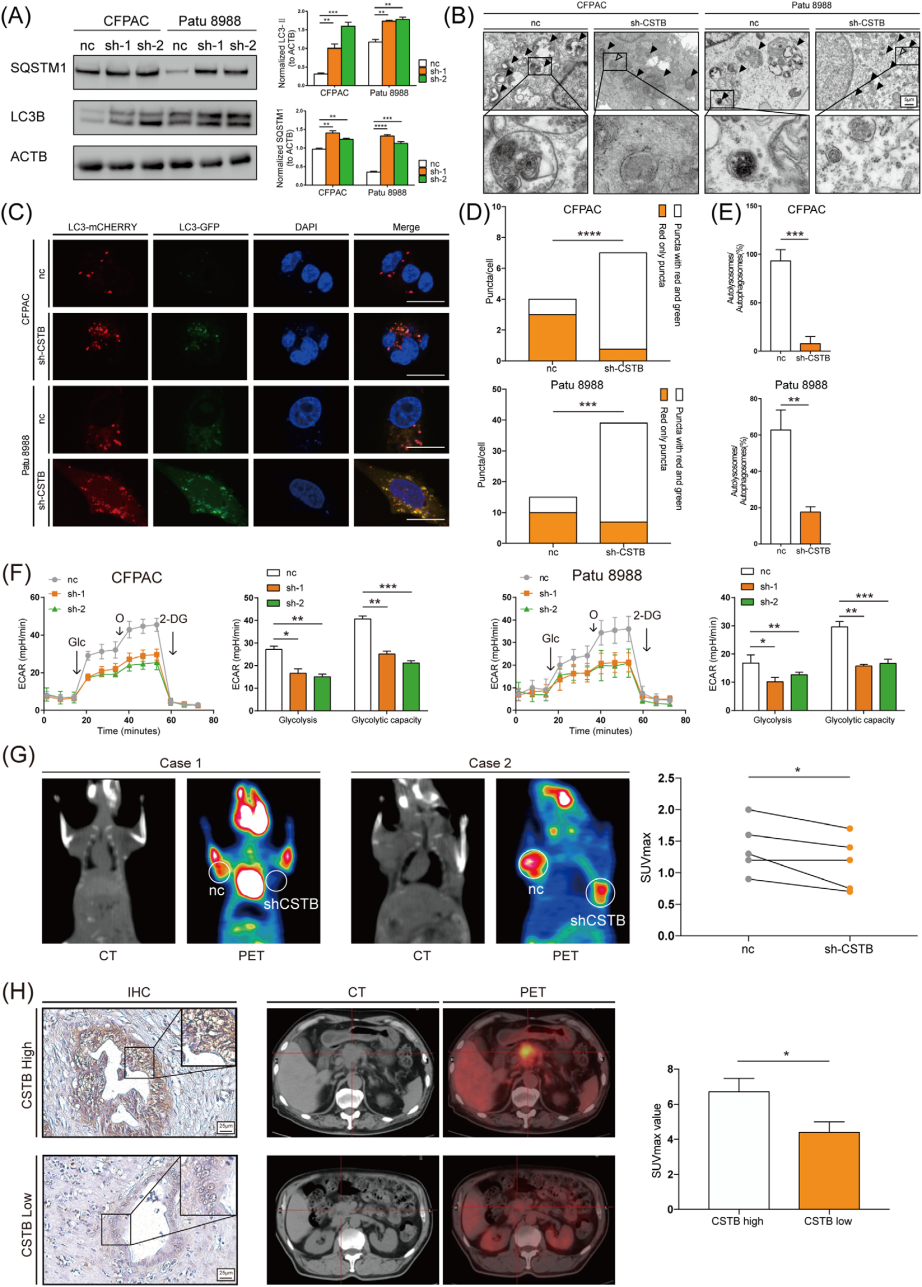
Autophagy flux and glycolysis are promoted by CSTB in pancreatic ductal adenocarcinoma (PDAC). (A) Representative images of SQSTM1, LC3B, and β‐Actin (loading control) immunoblots of control and sh‐CSTB CFPAC and Patu 8988 cells. (B) Transmission electron microscopy shows autolysosome (filled arrow) and autophagosome (hollow arrow) in control and sh‐CSTB‐1 CFPAC and Patu 8988 cells. (C) Representative images of control and sh‐CSTB‐1 CFPAC and Patu 8988 cells expressing mCherry–GFP–LC3B. (D,E) Quantification of LC3B puncta representing autophagosomes (yellow) and autolysosomes (red) in cells. (F) Extracellular acid rate detected by Seahorse analyser in both cell lines. (G) Graph of ^18^F‐FDG uptake in subcutaneous xenograft model. Control and sh‐CSTB‐1 Patu 8988 cells were used to establish subcutaneous xenograft model. (H) Representative images of CSTB expression in tumour tissues from PDAC patients who received preoperative 18F‐FDG PET/CT examination. The difference in the SUVmax value between CSTB‐high and CSTB‐low groups was analysed. Scale bar: 25 μm. *p < .05, **p < .01, ***p < .001, and ****p < .0001

To investigate the effect of CSTB on autophagy in PDAC, CSTB knockdown or overexpression was performed in three PDAC cell lines (knockdown: CFPAC‐1 and Patu8988; overexpression: Panc1), respectively (Figure S2A–F). Autophagosome markers LC3B‐II and SQSTM1 (p62) were accumulated in CSTB‐knockdown cells, suggesting that late‐stage autophagy was blocked (Figure 2A). CSTB overexpression showed the opposite result (Figure S2G). The results of transmission electron microscopy indicated elevated autophagosomes in CSTB‐knockdown cells (Figure 2B and Figure S2H). Furthermore, IF results showed that the fluorescence intensity of SQSTM1 was higher in CSTB‐knockdown cells than in control cells (Figure S2I). After a tandem mCherry‐enhanced green fluorescent protein‐LC3B expression plasmid was transfected into CFPAC and Patu8988, the bright green signals in cells suggested autolysosome disruption in CSTB‐knockdown cells, as GFP fluorescence quenched in an acidic lysosomal environment if autophagic flux was not obstructed (Figure 2C–E). Collectively, these results demonstrated that CSTB knockdown impaired the late stage of autophagy. Moreover, treatment with the autophagy inhibitor bafilomycin A_1_ (BAF) led to a significant decrease in the glucose consumption of PDAC cells, and CSTB overexpression could not restore glucose uptake (Figure S2J), which was in line with the notion that autophagy facilitated the recycling of glucose.

On the other hand, the roles of CSTB on glycolysis in PDAC was investigated. As expected, the hallmark of glycolysis genes was enriched in the high CSTB expression group in both TCGA and GSE 102238 (Figure S3A). The expression of CSTB was positively correlated with several key enzymes involved in glycolysis (Figure S3B). CSTB knockdown or overexpression significantly decreased or increased the expression levels of glycolysis‐related genes (S3C‐S3D). To further investigate the effect of CSTB on PDAC cells, the extracellular acid rate (ECAR) and oxygen consumption rate were measured by Seahorse XF Analyzers. CSTB knockdown led to decreased glycolysis and impaired glycolytic capacity, while there was no significant change in ATP production nor maximal respiration (Figure 2F and Figure S3E). These results suggested that CSTB increased the glycolytic portion of glucose metabolism in PDAC cells. Moreover, a similar conclusion was drawn in the lactate production, glucose uptake, and ATP generation assays (Figure S3F–H). Consistently, overexpression assays showed opposite results (Figure S3I–L). To confirm the role of CSTB in glycolysis by in vivo experiments, positron emission tomography (PET)/computed tomography (CT) imaging with ^18^F‐FDG was performed in a subcutaneous xenograft mouse model. SUVmax was significantly lower in tumours induced by CSTB‐knockdown cells (Figure 2G). Moreover, a cohort of 27 PDAC patients in Ruijin Hospital who underwent preoperative ^18^F‐FDG PET/CT scans was analysed. Higher expression levels of CSTB exhibited a higher SUVmax in clinical samples (Figure 2H). To check whether the effect of CSTB on glycolysis depended on autophagy, glycolysis‐related function assays were conducted with or without BAF treatment. As shown in Figure S3M,N, BAF compromised the pro‐glycolysis effect of CSTB. In addition, lactate production and ATP generation assays showed similar results (Figure S3O,P). Taken together, these results demonstrates that CSTB promotes glycolysis by increasing autophagy in PDAC.

## 6 CSTB promotes cell proliferation in PDAC

**FIGURE 3:**
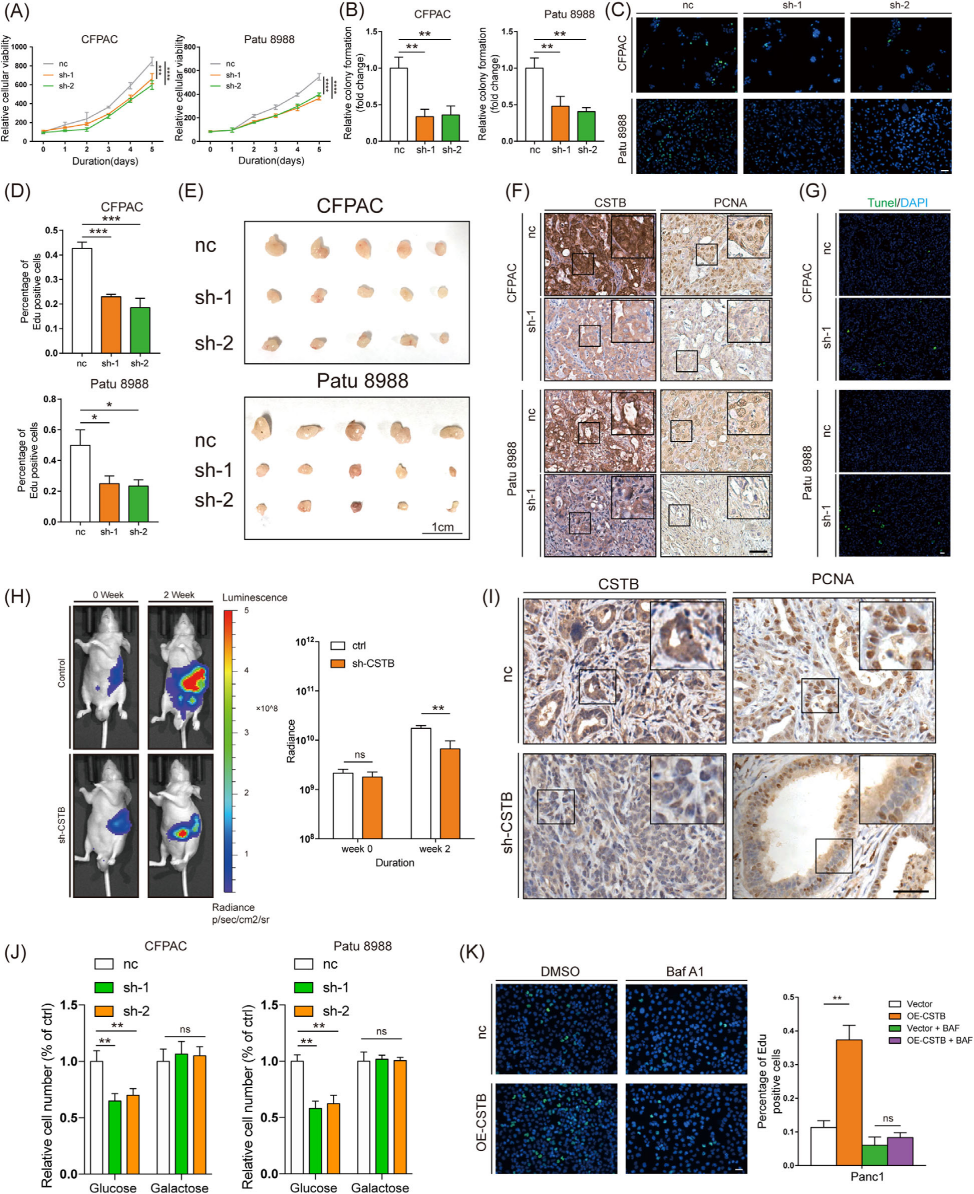
CSTB contributes to cell proliferation through glycolysis and autophagy. Cell proliferation of pancreatic cancer cells were measured by CCK8 (A), colony formation (B), and EdU assay (C,D) using control and sh‐CSTB CFPAC and Patu 8988 cells. (E) The effects of CSTB knockdown on the growth of subcutaneous pancreatic ductal adenocarcinoma (PDAC) xenografts. (F) Representative IHC images of CSTB and PCNA from subcutaneous PDAC xenografts samples. (G) Representative IF images of TUNEL from subcutaneous PDAC xenografts samples. (H) Representative bioluminescence photograph of mice orthotopically implanted with luciferase‐expressing Patu 8988 cells with or without sh‐CSTB‐1 transfected. (I) Representative IHC images of CSTB and PCNA from orthotopic pancreatic ductal adenocarcinoma xenografts samples. (J) Cell proliferation of control and sh‐CSTB CFPAC and Patu 8988 cells treated with or without galactose. (K) EdU assay of control and OE‐CSTB Panc1 cells treated with or without BAF. Scale bar: 25 μm. *p < .05, **p < .01, ***p < .001, and ****p < .0001

Glycolysis and autophagy enable cancer cells to acquire and recycle nutrients in a manner that is conducive to proliferation.^5^, ^11^, ^12^, ^39^ In vitro functional assays including CCK8, colony formation, and EdU assays demonstrated that CSTB knockdown attenuated the proliferation of PDAC cells (Figure 3A–D and Figure S4A). As expected, CSTB overexpression significantly enhanced cell proliferation (Figure S4B–D). Two animal models were used to further investigate in vivo tumorigenicity of CSTB. The average volume of tumours induced by CSTB knockdown was significantly smaller than that of the controls in the subcutaneous xenograft model (Figure 3E and Figure S4E). IHC staining showed that a stronger PCNA intensity was observed in tumours induced by higher CSTB‐expressing cells (Figure 3F). Furthermore, CSTB knockdown decreased the apoptotic index, as assessed by the TUNEL assay (Figure 3G and Figure S4F). In the pancreatic orthotopic tumour formation model, tumours induced by CSTB‐knockdown cells showed lower bioluminescent emission compared with the control group (Figure 3H), and the expression pattern of proliferating cell nuclear antigen (PCNA) in orthotopic tumours was consistent with that in subcutaneous tumours (Figure 3I).

To further investigate whether CSTB promoted cell proliferation depending on glycolysis and autophagy, an alteration to glucose (galactose), glycolytic inhibitor (2‐deoxy‐d‐glucose, 2‐DG), and autophagy inhibitor (BAF) were added into cell culture individually. Both galactose and 2‐DG could compromise the difference in the capacity of cell proliferation between CSTB knockdown and control cells (Figure 3J and Figure S4G). Also, BAF abolished the pro‐proliferative effects of CSTB overexpression (Figure 3K and Figure S4H).

## 7 CSTB increases autophagic flux via enhancing the proteolytic activity of cathepsin B

**FIGURE 4:**
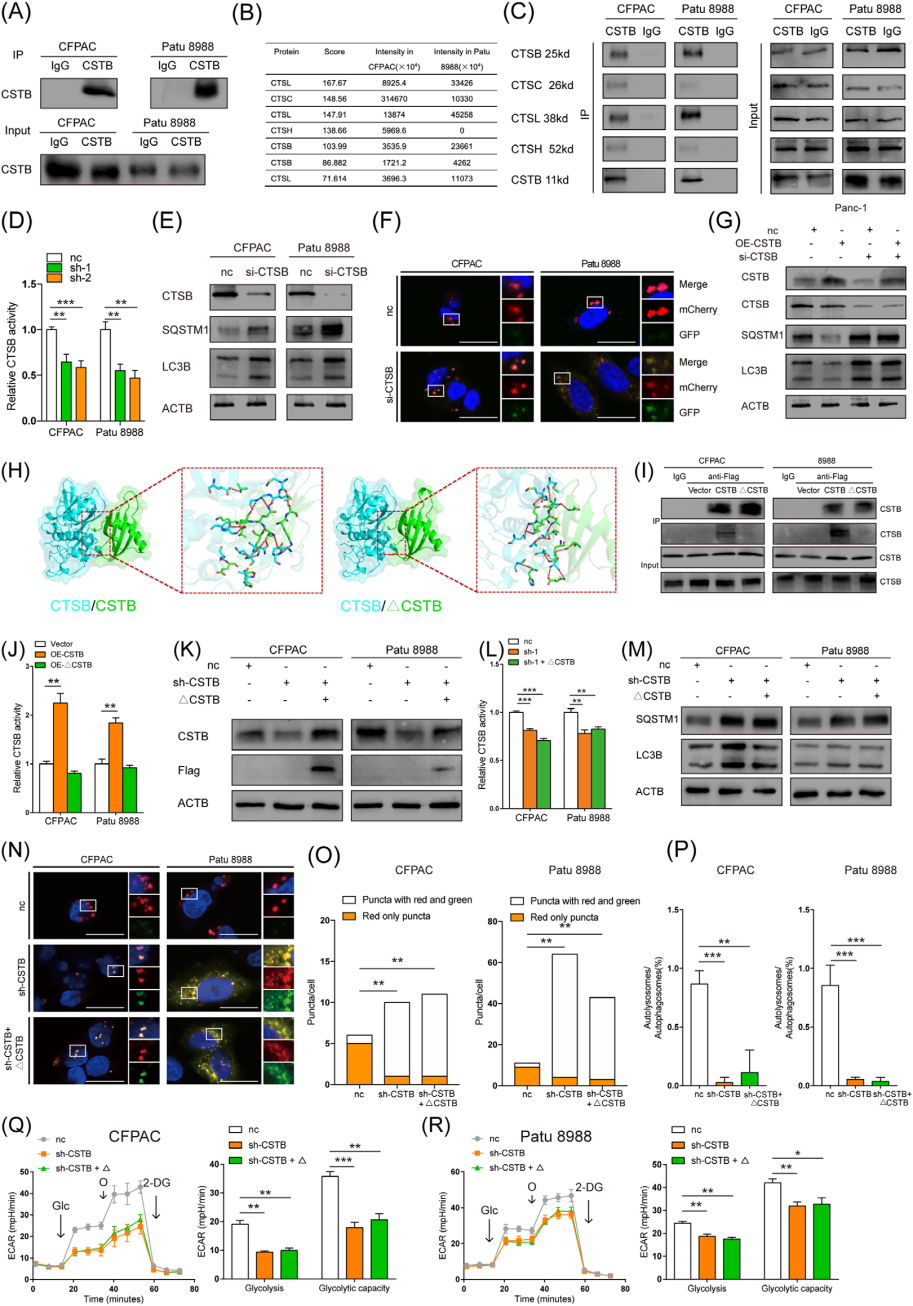
CSTB interacts with Cathepsin B (CTSB) to favour pancreatic ductal adenocarcinoma. (A) Immunoprecipitation of CSTB in CFPAC and Patu 8988. (B) Cathepsins interact with CSTB are indicated by LC/MS. (C) Western blot detected cathepsins in proteins that immunoprecipitated with CSTB. (D) Relative CTSB activity of control and sh‐CSTB CFPAC and Patu 8988 cells. (E) Immunoblots of CTSB, SQSTM1, and LC3B in control and si‐CTSB CFPAC and Patu 8988 cells. (F) Representative images of control and si‐CTSB CFPAC and Patu 8988 cells expressing mCherry–GFP–LC3B. (G) Immunoblots of CSTB, CTSB, SQSTM1, and LC3B in control and OE‐CSTB Panc1 cells transfected with or without si‐CTSB. (H) Left: Docking mode of CTSB/CSTB and hydrogen bonds (blue CTSB, green CSTB^WT^). Right: Docking mode of CTSB/△CSTB and hydrogen bonds (blue CTSB, green CSTB^G50E^). (I) Immunoblots of CSTB and CTSB in CFPAC and Patu 8988 cells transfected with Vector, CSTB, and mutant CSTB, respectively. (J) Relative CTSB activity of control and OE‐CSTB Panc1 cells transfected with or without mutant CTSB. (K) Immunoblots of CSTB and Flag in control and sh‐CSTB‐1 CFPAC and Patu 8988 cells transfected with or without mutant CTSB. (L) Relative CTSB activity of control and sh‐CSTB CFPAC and Patu 8988 cells transfected with or without mutant CTSB. (M) Immunoblots of SQSTM1and LC3B in control and sh‐CSTB‐1 CFPAC and Patu 8988 cells transfected with or without mutant CTSB. (N) Representative images of control and sh‐CSTB CFPAC and Patu 8988 cells expressing mCherry–GFP–LC3B transfected with or without mutant CSTB. (O,P) Quantification of LC3B puncta representing autophagosomes (yellow) and autolysosomes (red) in cells. (Q,R) Extracellular acid rate detected by Seahorse analyser in control and sh‐CSTB‐1 CFPAC and Patu 8988 cells transfected with or without mutant CSTB. Scale bar: 25 μm. *p < .05, **p < .01, ***p < .001, and ****p < .0001

To unveil the underlying mechanism of increased autophagic flux by CSTB, the key factors of autophagy (including Beclin1, ATG4, 5, 7, 16, and LAMP1, 2) were checked by western blotting (WB). Lysotracker staining was performed as well. However, these factors exhibited no obvious differences between CSTB knockdown and control cells (Figure S5A,B). As a member of the cystatin family, CSTB is crucial for modulating the proteolytic activity of its target cysteine proteases (cathepsin), which are major lysosomal proteases. Thus, we focused on the members of cathepsin family interacting with CSTB. LC‐MS against the products of Co‐IP with CSTB antibody was performed to identify potential candidates, which were further confirmed by WB (Figure 4A–C). Among them, only CTSB enzyme activity was decreased in CSTB‐knockdown cells (Figure 4D and Figure S5C). It has been reported that CTSB deficiency in the mouse pancreas impairs autophagy.^40^ Similar to CSTB knockdown, CTSB silencing also led to impaired autophagic flux (Figure 4E,F and Figure S5D,E). Once CTSB was silenced, CSTB overexpression failed to increase autophagic flux (Figure 4G). Moreover, CTSB silencing also abolished CSTB‐mediated glycolysis and cell proliferation in PDAC (Figure S5F–K). Thus, we concluded that CSTB promoted autophagy and glycolysis by interacting with CTSB and enhancing its proteolytic activity.

The highly conserved QVVAG motif of CSTB in the first beta‐hairpin loop is vital to interact with the target cathepsins at the crystal structure level.^41^, ^42^ Amino acid changes in the QVVAG motif can strikingly affect the interaction and subsequently attenuate the inhibitory effect. A C.149 G > A CSTB mutant plasmid was constructed and generated a mutant CSTB protein (△CSTB) in which glutamic acid at codon 50 was replaced by glycine (P.G50E). The highly conserved QVVAG papain‐binding region was altered in △CSTB. The docking mode of CTSB/CSTB and CTSB/△CSTB was then constructed using the calculated ZDOCK score. A higher ZDOCK score indicates a more effective binding affinity.^43^ Of note, CTSB/CSTB formed 15 hydrogen bonds and 12 hydrogen bonds in CTSB/△CSTB, respectively. The contact surface area of CTSB/CSTB was 1380.3 Å^2^ and of CTSB/△CSTB was 1196.8 Å2 (Figure 4H). Moreover, IP assays confirmed that △CSTB with an altered QVVAG motif possessed a weaker capacity to bind to CTSB than wild type CSTB in two PDAC cell lines (Figure 4I). Impaired proteolytic activity of CTSB could not be restored by △CSTB in CSTB‐knockdown cell lines (Figure 4J–L). Moreover, △CSTB overexpression in CSTB‐knockdown cells failed to restore the obstructed autophagic flux (Figure 4 M–P). Also, glycolysis could not be recovered by △CSTB overexpression (Figure 4Q,R). Collectively, we can conclude that the QVVAG motif is indispensable for CSTB to interact with CTSB and enhance its proteolytic activity.

## 8 CSTB competed with CSTC to interact with cathepsin B

**FIGURE 5:**
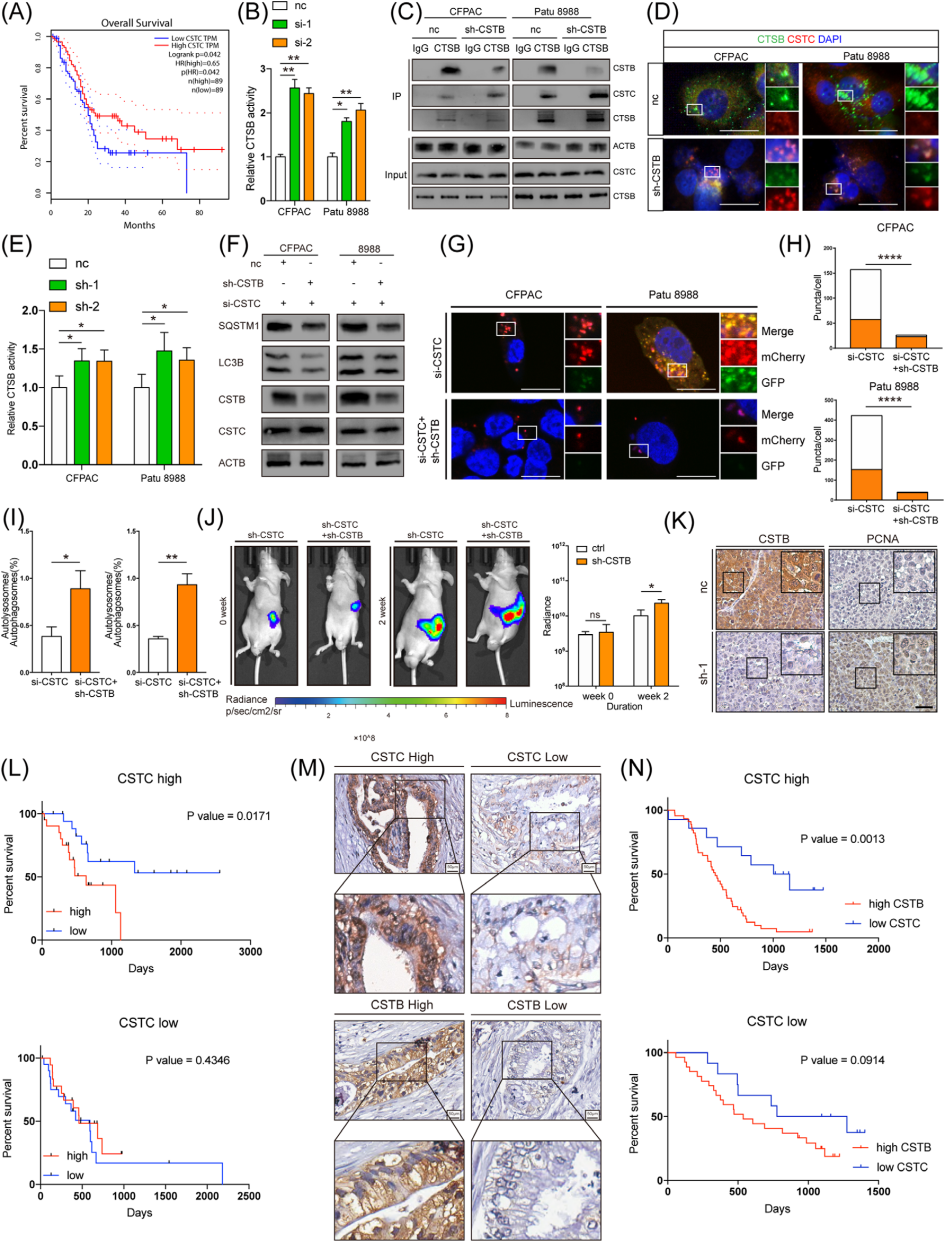
CSTB interacts with Cathepsin B (CTSB) to prevent it from binding to CSTC. (A) Kaplan–Meier curves show that patients with high CSTC were accompanied with better overall survival (OS). (B) Relative CTSB activity of control and si‐CTSB CFPAC and Patu 8988 cells. (C) Immunoblots of CSTB, CSTC and CTSB in immunoprecipitate of CTSB in control and sh‐CSTB‐1 CFPAC and Patu 8988 cells. (D) IF staining for CTSB (green) and CSTC (red) in control and sh‐CSTB‐1 CFPAC and Patu 8988 cells. (E) Relative CTSB activity of control and sh‐CSTB CFPAC and Patu 8988 cell with prior CTSB silencing. (F) Immunoblots of SQSTM1, LC3B, CSTB, and CSTC in control and sh‐CSTB‐1 CFPAC and Patu 8988 cells transfected with si‐CSTC. (G) Representative images of control and sh‐CSTB‐1 CFPAC and Patu 8988 cells with prior CTSB silencing expressing mCherry‐GFP–LC3B. (H,I) Quantification of LC3B puncta representing autophagosomes (yellow) and autolysosomes (red) in cells. (J) Representative bioluminescence photograph of mice orthotopically implanted with luciferase‐expressing and CSTC‐silencing Patu 8988 cells with or without sh‐CSTB‐1. (K) Representative IHC images of CSTB and PCNA from orthotopic pancreatic ductal adenocarcinoma (PDAC) xenografts samples. (L) Kaplan–Meier analysis of CSTB in patients with high CSTC expression (upper, 45) and patients with low CSTC expression (lower, 45) in TCGA database. (M) Representative images of CSTC and CSTB in Ruijin TMA spotted with human PDAC tissue cores. (N) Kaplan–Meier analysis of CSTB in patients with high CSTC expression (upper, 59) and patients with low CSTC expression (lower, 40) in the Ruijn cohort. Scale bar: 25 μm. *p < .05, **p < .01, ***p < .001, and ****p < .0001

Assuming that CSTB is a protease inhibitor, it is bewildering that CSTB in PDAC cells contributed to increased CTSB activity. More than 10 types of cystatins exist in mammalian cells, and their inhibitory intensity varies. Compared to CTSL, S, and H, CSTB showed decreased activity toward CTSB by approximately 3‐4 orders of magnitude.^44^, ^45^ Thus, we hypothesised that CSTB might protect CTSB from other cystatins with stronger protease activity. Among the types of cystatins, only CSTB is associated with poorer prognosis, whereas CSTC is associated with better prognosis in PDAC samples from TCGA (Figure 5A,H). Moreover, CSTC is recognised as the most potent inhibitor of cathepsins.^46^ Therefore, the effect of CSTC on the proteolytic activity of CTSB was investigated. Not surprisingly, CSTC knockdown significantly enhanced CTSB activity in PDAC cells (Figure 5B). CSTC was increased in the immunoprecipitate against CTSB of CSTB‐knockdown cells compared with controls. (Figure 5C). This suggested that CSTB competed with CSTC to interact with CTSB. Furthermore, IF assays showed that the co‐localisation of CSTC and CTSB was increased in CSTB‐knockdown cells compared to control cells (Figure 5D).

Next, we investigated whether CSTC abolished the effect of CSTB on PDAC progression. Interestingly, CSTC silencing reversed the impact of CSTB on CTSB. Knockdown efficiency was shown in Figure S6A. Unlike in parental PDAC cells, CSTB knockdown led to the increased activity of CTSB in cells with prior CSTC silencing (Figure 5E). In addition, the effect of CSTB on autophagy, glycolysis and proliferation was also switched after CSTC silencing treatment (Figure 5F–I and Figure S6B–E). Similar results were obtained from in vivo experiments (Figure 5J,K). By analysing the expression pattern of CSTB and CSTC in our in‐house cohort as well as TCGA, we strikingly found that CSTB was significantly associated with prognosis in the CSTC high‐expression group, while no association was observed in the CSTC low‐expression group of PDAC samples in both cohorts. (Figure 5L–N).

## 9 H3K27 acetylation in CSTB promoter region facilitates SP1 transcription of CSTB

**FIGURE 6:**
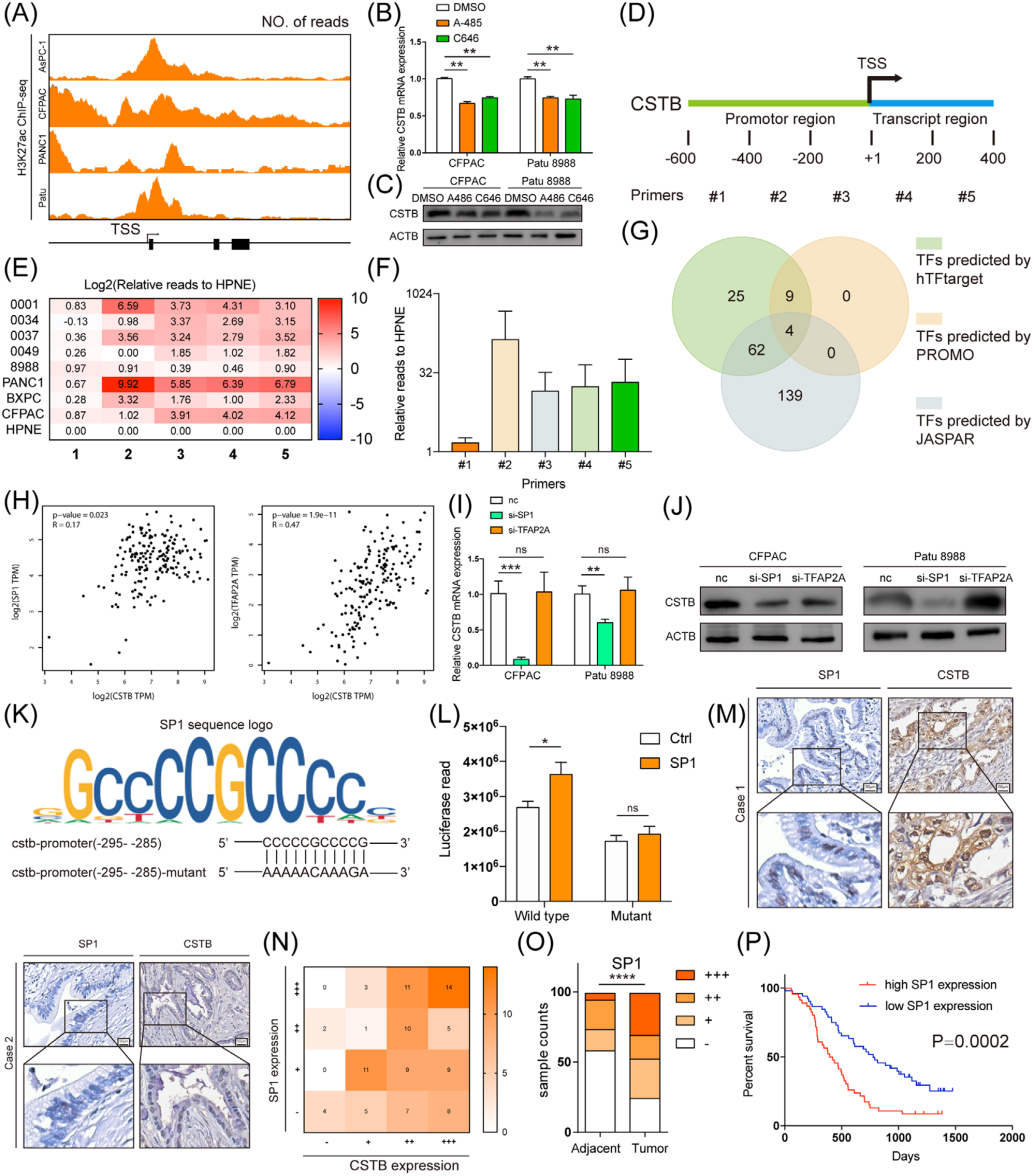
CSTB is up‐regulated by SP1 in pancreatic ductal adenocarcinoma (PDAC). (A) Predicted H3K27ac binding site in CSTB promoter from UCSC genome browser. Real‐time expression of CSTB in CFPAC and Patu 8988 treated with DMSO, A485 or C646 in RNA (B) and protein (C) levels. (D) Diagram showing the designed corresponding primers. (E) Heatmap showing relative H3K27ac reads in five segments of CSTB promoter in nine cell lines. (F) Average H3K27ac reads of CSTB promoter in eight PDAC cell lines, relative to HPNE. (G) Venn diagram showing TF screening strategy. (H) Analysis of correlation between CSTB and SP1 (left) and TFAP2A (right) in TCGA. Real‐time expression of CSTB in CFPAC and Patu 8988 transfected with si‐SP1 or si‐TFAP2A in RNA (I) and protein (J) levels. (K) The sequence logo graph manifested the canonical binding site of SP1 predicted by JASPAR. (L) Luciferase activity in HEK‐293T cells co‐transfected with SP1 or scramble sequence and plasmid. (M) Two representative IHC staining images of SP1 and CSTB in Ruijin TMA. (N) Correlation between CSTB and SP1 in Ruijin TMA. (O) Respective sample counts in tumour tissues and adjacent tissues. (P) Kaplan–Meier analysis of overall survival rate related to the expression of SP1 in Ruijin TMA *p < .05, **p < .01, ***p < .001, and ****p < .0001

Since genes involved in autophagy and glycolysis are more likely to be upregulated in hypoxia environment,^47^ we then explored if hypoxia is able to alter the expression of CSTB. However, cells cultured in hypoxia condition did not exhibit significant higher expression of CSTB compared with control cells (Figure S7A). Changes in DNA methylation are partly responsible for the mRNA expression patterns linked to hypoxia.^47^ According to TCGA, CSTB promoter methylation profile did not show evident alterations between tumours and normal tissues as well (Figure S7B). Histone modifications have been proved to act as a key role in the control of the cancer gene. Among these, H3K27 acetylation (H3K27ac) is a recognised indicator of the activation of transcription. To further investigate the mechanism of CSTB expression regulation in PDAC, we explored the transcriptional modification of CSTB in the UCSC genome browser. The H3K27ac binding site was found to be enriched in the promoter region of CSTB (Figure 6A). Two PDAC cell lines with increased CSTB expression were treated with two histone acetyltransferase inhibitors, A‐485 and C646; thereafter, the expression of CSTB was decreased at both mRNA and protein levels (Figure 6B,C). The H3K27 acetylation enriched region near the transcription start site of CSTB was divided into five segments with a length of approximately 200 bp, and the corresponding primers were designed (Figure 6D). ChIP‐PCR data against H3K27ac in eight PDAC cell lines and HPNE indicated that higher H3K27ac levels were observed in cancer cells than in HPNE (Figure 6E). Among the five segments, the #2 segment had the highest number of acetylation reads (Figure 6F).

Moreover, potential transcription factors (TFs) were predicted using three databases (hTFtarget, PROMO, and JASPAR) based on the base arrangement of #2 segments. Although four TFs (SP1, TFAP2A, GATA2, and PAX5) were screened out, only the expression levels of SP1 and TFAP2A were positively correlated with that of CSTB in PDAC samples from TCGA (Figure 6G,H). Moreover, SP1 silencing resulted in decreased expression of CSTB (Figure 6I,J). Therefore, SP1 was selected for further investigation. The sequence of −295 to −285 in segment #2 was predicted as a canonical binding site of SP1. The luciferase reporter assays further demonstrated that SP1 activated the transcription of CSTB by binding to the predicted binding site (Figure 6K,L). Furthermore, a positive correlation between SP1 and CSTB was observed in PDAC clinical samples (Figure 6 M,N). SP1 was upregulated in PDAC and the upregulation was associated with poor prognosis (Figure 6O,P). Collectively, these results suggested that high H3K27ac levels in the promoter region and SP1 upregulation contributed to the high expression of CSTB in PDAC.

## 10 CSTB facilitates PDAC growth in patient‐derived xenograft mice model

**FIGURE 7:**
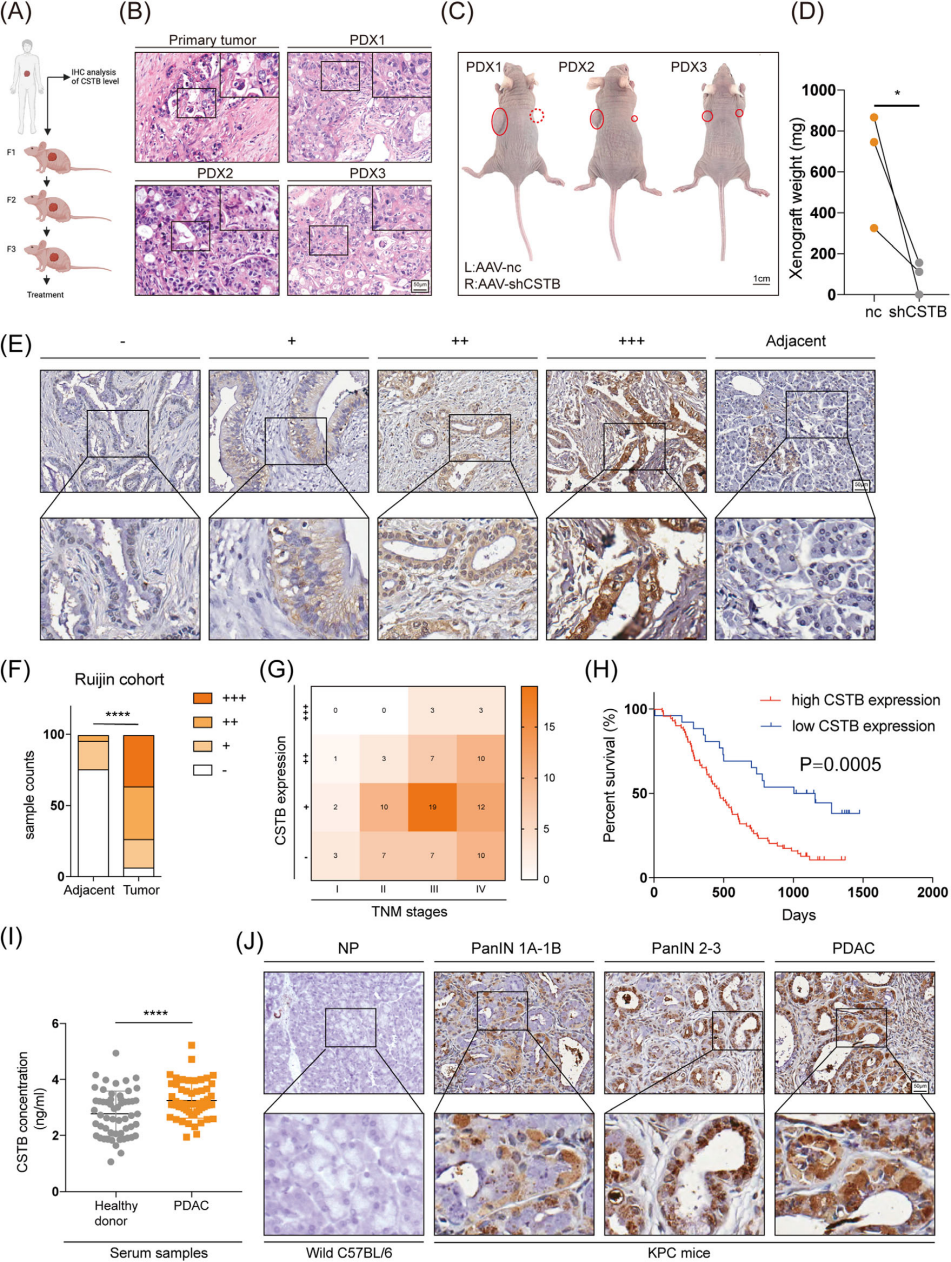
CSTB is significantly upregulated in pancreatic cancer and has clinical significance. (A) Diagram showing the PDX procedure and dosing regime in the therapy model; mice were given intratumoral injections of AAV twice a week for 4 weeks. (B) Representative HE images of primary tumour (F0) and PDX (F3) tumour tissue samples. (C) Gross view of PDX model 4 weeks after AAV injection. Left xenografts were treated with scramble AAV while right xenografts were treated with CSTB‐targeting AAV. (D) Xenografts’ weight was evidently decreased when treated with CSTB‐targeting AAV. (E) Representative IHC images of CSTB expression in Ruijin TMA spotted with human PDAC tissue cores. (F) Respective sample counts in tumour tissues and adjacent tissues. (G) Heatmap shows the counted numbers of four levels of CSTB expression and its correlation with TNM classification. (H) Kaplan–Meier analysis of overall survival rate related to the expression of CSTB expression in Ruijin TMA. (I) CSTB concentration in the serum of 56 healthy donors and 57 PDAC patients. (J) Representative IHC images of CSTB in different stages of PDAC progression in KPC mice. *p < .05, **p < .01, ***p < .001, and ****p < .0001

To test the therapeutically benefit of CSTB on PDAC patients, we generated PDAC PDX murine models using tumour samples from three patients (Figure 7A). HE staining was first performed to confirm the pathological diagnosis (Figure 7B). AAV carrying CSTB‐targeting shRNA or scramble shRNA were intratumorally injected twice a week for 4 weeks, starting from week 8 after transplantation. Expression of CSTB was evidently decreased in knockdown group according to IHC (Figure S8A). As shown in Figure 7C,D, CSTB knockdown resulted in a remarkable decrease in tumour growth.

## 11 Expression pattern and clinical significance of CSTB in PDAC progression

Clinical samples in tissue microarray (TMA) were separated into four groups based on the IHC staining ratio and intensity to examine the clinical importance of CSTB in PDAC (Figure 7E). In comparison to surrounding tissues, tumour tissues showed a higher expression of CSTB (Figure 7F). Poor overall survival and advanced tumour stages were strongly correlated with high expression of CSTB (Figure 7G,H). As shown in Figure S8B, CSTB also serves as a prognostic marker representing shorter survival time despite the smoking history, vascular invasion status, CA125 level, and CEA level. Chi‐square analysis indicated that the expression of CSTB was significantly correlated with tumour differentiation (Table S2). Univariate and multivariate analyses showed that CSTB expression was an independent prognostic factor for the overall survival of patients with PDAC (Table S3). Moreover, the concentration of CSTB was detected in serum samples from 57 PDAC patients and 56 healthy donors. A significantly higher level of CSTB was observed in the serum of PDAC patients (Figure 7I), which was consistent with previous studies of several other types of cancer.^19^, ^48^, ^49^, ^50^, ^51^ The Kras^G12D/+^/Trp‐53^R172H/+^/Pdx1‐Cre (KPC) mice were utilised to examine the dynamic alterations and expression of CSTB in PDAC formation and progression. These mice exhibit greatly accelerated PanIN development and well‐differentiated PDAC. Interestingly, the expression of CSTB gradually increased as pancreatic tissues underwent PanIN and were finally transformed into PDAC (Figure 7J).

## 12 DISCUSSION

Although autophagy is recongnised as a dynamic recycling system that provides nutrition substrates and energy for cell proliferation and homeostasis,^5^ the detailed role of autophagy in cancer remains largely unknown. A tumour‐suppressive role of autophagy has been reported and seems to be of great importance in liver cancer.^52^ On the other hand, cancer cells generally have a heightened metabolic demand for energy source and building blocks for proliferation. Especially, PDAC cells trapped in a hostile TME with high desmoplasia and poor vascularity are ‘addictive’ to autophagy for survival.^5^ Autophagy plays a role in scavenging lysosome nutrient to maintain cellular homeostasis in PDAC cells.^53^, ^54^ Yang et al. indicated pancreatic cancer cells, particularly those with Ras mutation relied heavily on autophagy for tumour growth.^55^ Besides Ras mutation, *p53* status also determines the role of autophagy in PDAC development.^8^ Given the role of autophagy in PDAC progression, autophagy suppression could be a strategy for cancer treatment. To date, multiple clinical trials have demonstrated that autophagy inhibitors can improve the survival of PDAC.^2^, ^7^, ^8^ Consistent with previous studies, we found autophagy was enhanced in PDAC samples from TCGA compared with normal pancreatic tissue from GTEx.

Moreover, the autophagy and glycolysis level were positively correlated with each other in all the enrolled PDAC datasets. Unlike autophagy, glycolysis has been generally considered to be a pro‐tumour role in most types of cancer.^56^, ^57^ By further bioinformatic analysis, we surprisingly found that autophagy signature was still positively correlated with glycolysis signature in samples with autophagy inhibitor. However, no significant correlation between them was observed in samples with glycolysis inhibitor treatment (data not shown). These results suggested there was a regulatory role of autophagy on glycolysis in PDAC, but impaired glycolysis failed to regulate the autophagy level. Although some previous studies have indicated that autophagy could provide glycolysis with recycled glucose, the links between autophagy and glycolysis are still obscure.

In our study, we identified CSTB as a hub gene in the two biological processes of PDAC. CSTB is a member of the cystatin superfamily and inhibits cathepsins and papain by forming tight complexes.^14^, ^15^ As a main constituent of cellular protease, dysregulation of cathepsins is commonly accompanied with impaired proteolytic activity, which is required in many stages of tumour progression.^58^, ^59^, ^60^, ^61^ Aberrant expression of CSTB has been reported in several types of cancers. In our study, CSTB emerged as a key factor positively correlated with both glycolysis and autophagy in PDAC. Further in vivo and in vitro experiments confirmed that CSTB knockdown led to impaired autophagic flux, decreased glycolysis, and cell proliferation in PDAC. The mechanism of CSTB in PDAC tumorigenicity is due to the enhanced proteolytic activity of CTSB. As a member of cathepsin, CTSB is indispensable for the maintenance of cellular proteostasis by turning over substrates of autophagy. Similar to our findings, Iwama's work also showed that the deficiency of CTSB in the mouse pancreas results in inhibited autophagy.^40^ The mutated key motif of CSTB decreased the interaction between CSTB and CTSB, leading to compromised autophagy and glycolysis. These results reinforced that CSTB contributed to PDAC progression via CTSB. Although members of the cystatin family are widely considered as protease inhibitors, their working intensity on cathepsins varies. For instance, CSTB inhibits cathepsin H, L, S, and C at picomolar concentrations and CTSB at nanomolar concentrations.^16^, ^46^ In this study, we found that CSTB competed with CSTC to bind to CTSB. CSTC is considered one of the most powerful cathepsin inhibitors, and its roles in tumour progression are controversial.^46^ CSTC expression is associated with a better prognosis and acts as a tumour suppressor in PDAC. Previous studies also demonstrated that its suppressive role on tumours was mainly due to its inhibitory activity against cathepsins, especially CTSB.^62^, ^63^ Upon the CSTC silencing, the effects of CSTB on autophagy, glycolysis, and cell proliferation in PDAC cells were reversed in our study.

The clinical significance of CSTB in PDAC development and progression was also determined in KPC mouse model and PDAC clinical samples. Its expression was gradually enhanced in normal pancreas, PanIN, and PDAC tissues. PET imaging indicated that the SUVmax value from PET/CT was higher in PDAC patients with stronger CSTB staining, which confirmed the positive correlation between CSTB and glucose uptake at the clinical level. IHC staining results of PDAC TMA showed that higher expression of CSTB was associated with key clinicopathological features. Moreover, CSTB in serum could be used as a potential marker to distinguish PDAC patients from healthy people, which was consistent with previous studies of several other types of cancer.^19^, ^48^, ^49^, ^50^, ^51^ As the presence of CSTC is vital for the role of CSTB in tumour progression, CSTB was significantly associated with the prognosis in the CSTC high‐expression group rather than the CSTC low‐expression group in our in‐house PDAC cohort and TCGA.

## 13 CONCLUSIONS

**FIGURE 8:**
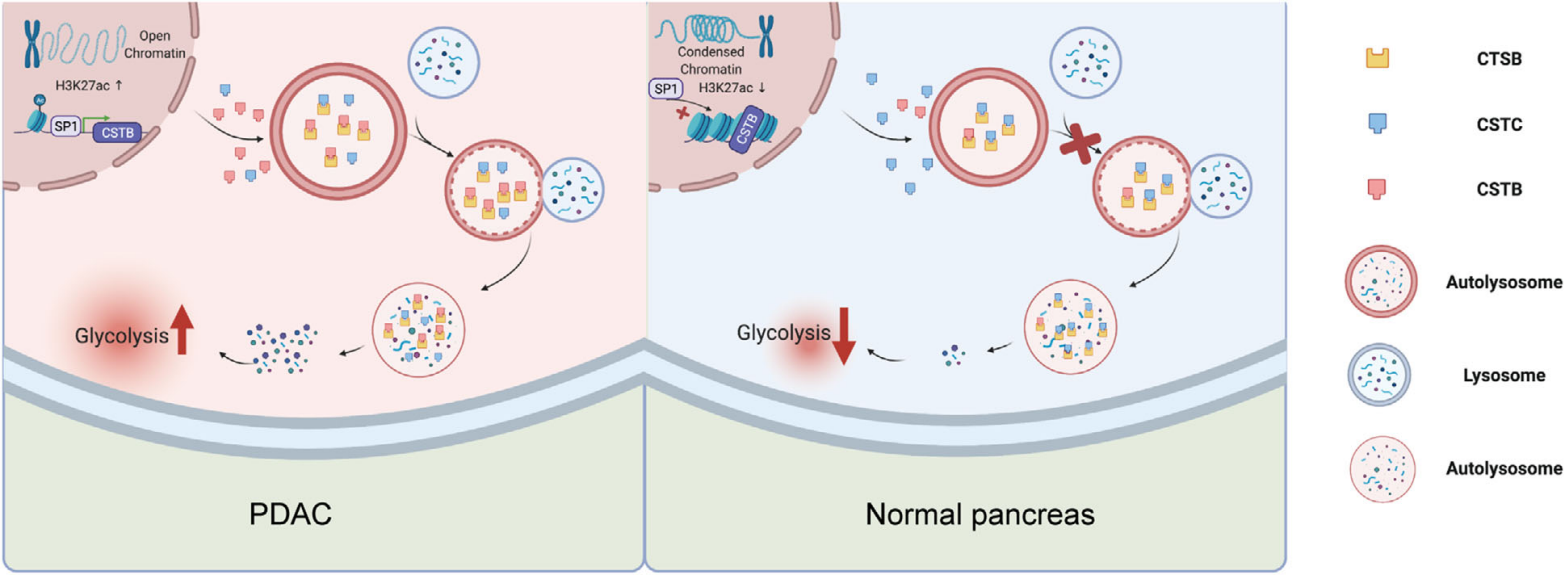
Graphical abstract illustrates up‐regulated by SP1, CSTB protects CTSB from CSTC and subsequently favour the autophagy flux and glycolysis in pancreatic ductal adenocarcinoma cells

Our study demonstrated CSTB should be a driver gene in PDAC development and progression as it promotes autophagic flux by competing with CSTC to bind to CTSB and provides substrates for glycolysis in PDAC. High H3K27ac levels in the promoter region and SP1 upregulation contributed to the high expression of CSTB (Figure 8).

## CONFLICT OF INTEREST

The authors declare no conflict of interest.

## Supporting information

Supporting Information

Supporting Information

Supporting Information

Supporting Information

Supporting Information

Supporting Information

Supporting Information

Supporting Information

Supporting Information

Supporting Information

Supporting Information

Supporting Information

Supporting Information

Supporting Information

Supporting Information

Supporting Information
