# Supplementary figures and images for: Cystatin B increases autophagic flux by sustaining proteolytic activity of cathepsin B and fuels glycolysis in pancreatic cancer: CSTB orchestrates autophagy and glycolysis in PDAC
Source: Clin Transl Med. 2022 Dec 10;12(12):e1126. doi: 10.1002/ctm2.1126 (PMC9736795; doi:10.1002/ctm2.1126)

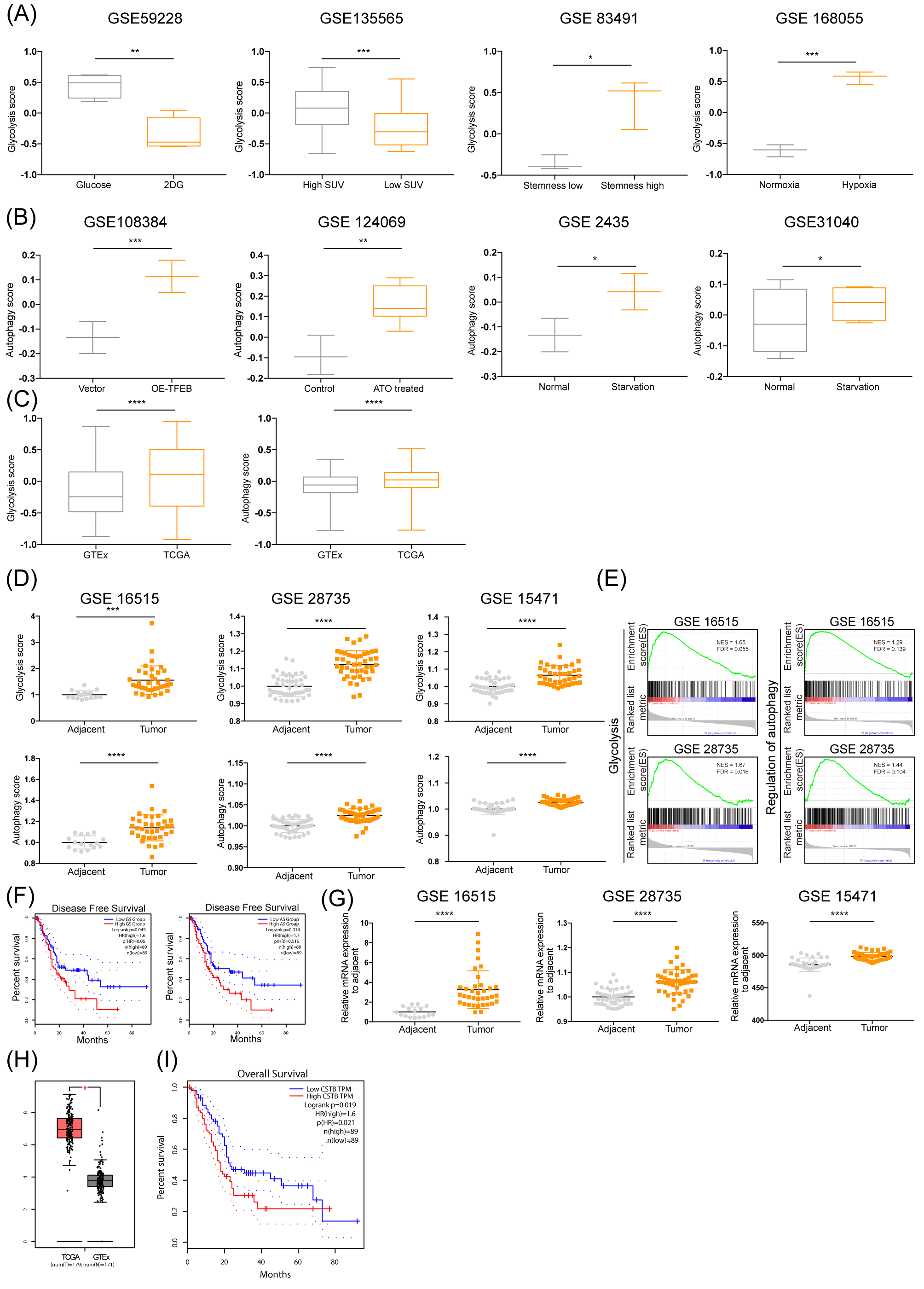

Supplement: Supplementary file 1 — Supporting Information [file CTM2-12-e1126-s009.tif]

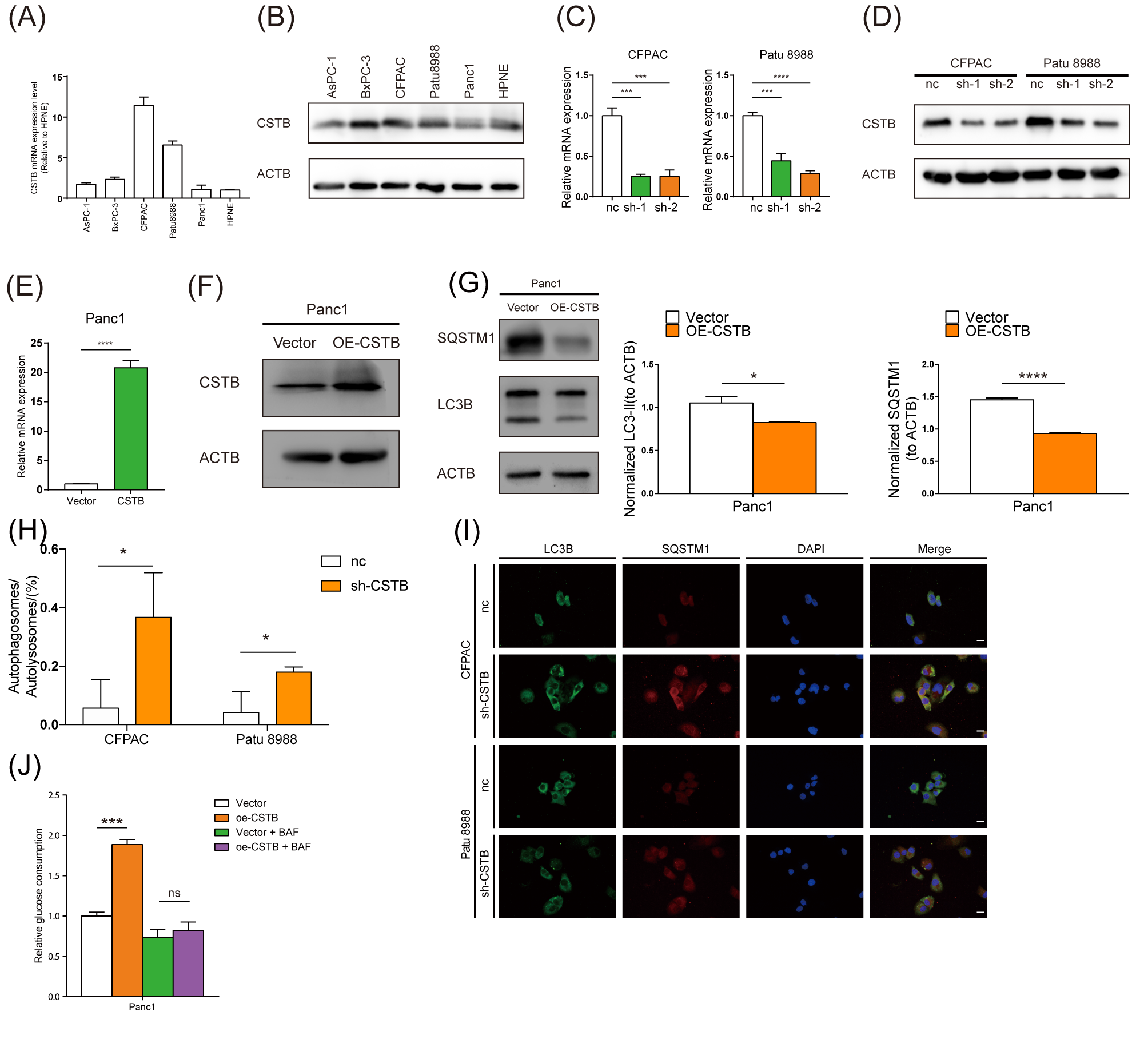

Supplement: Supplementary file 2 — Supporting Information [file CTM2-12-e1126-s013.tif]

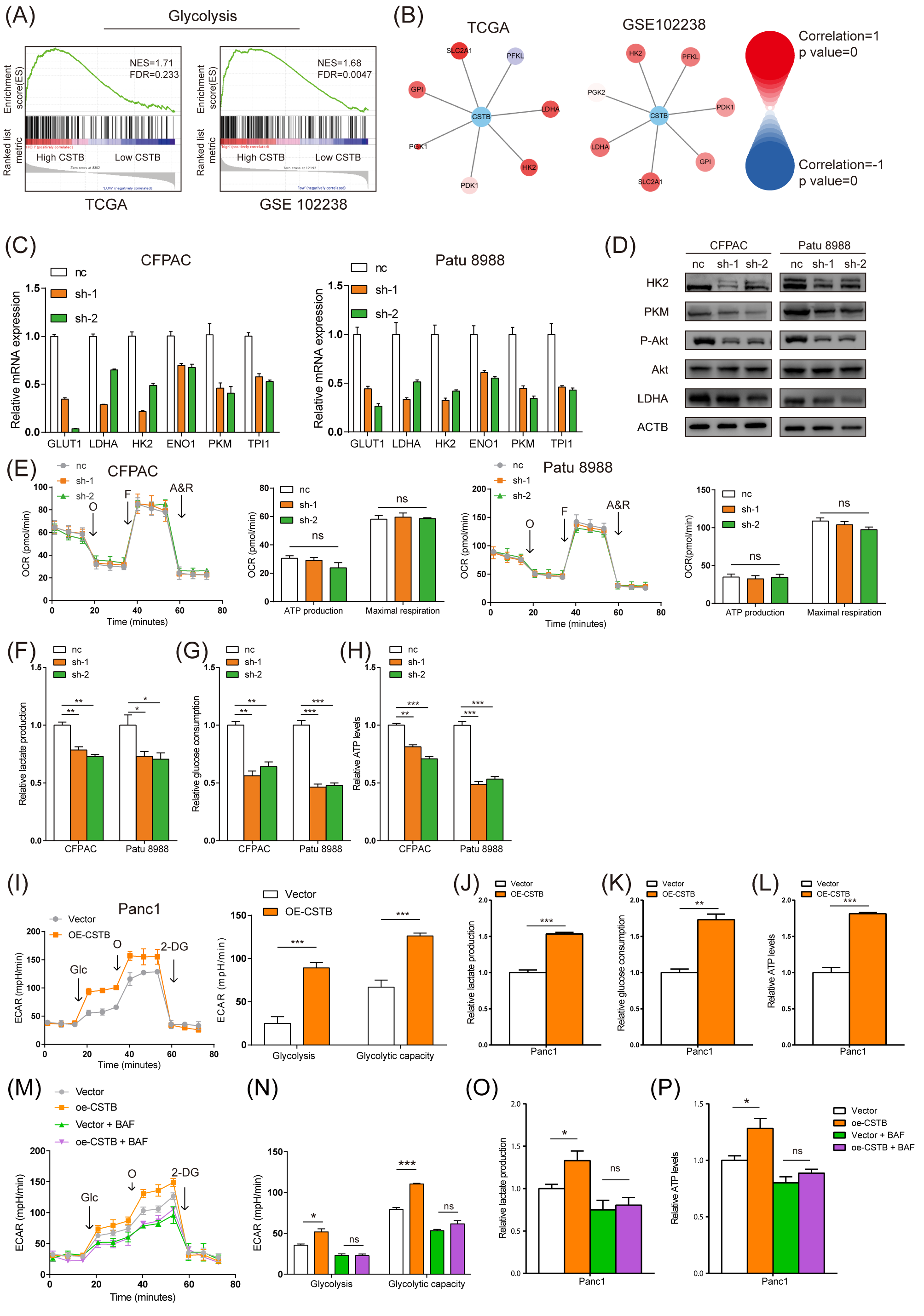

Supplement: Supplementary file 3 — Supporting Information [file CTM2-12-e1126-s002.tif]

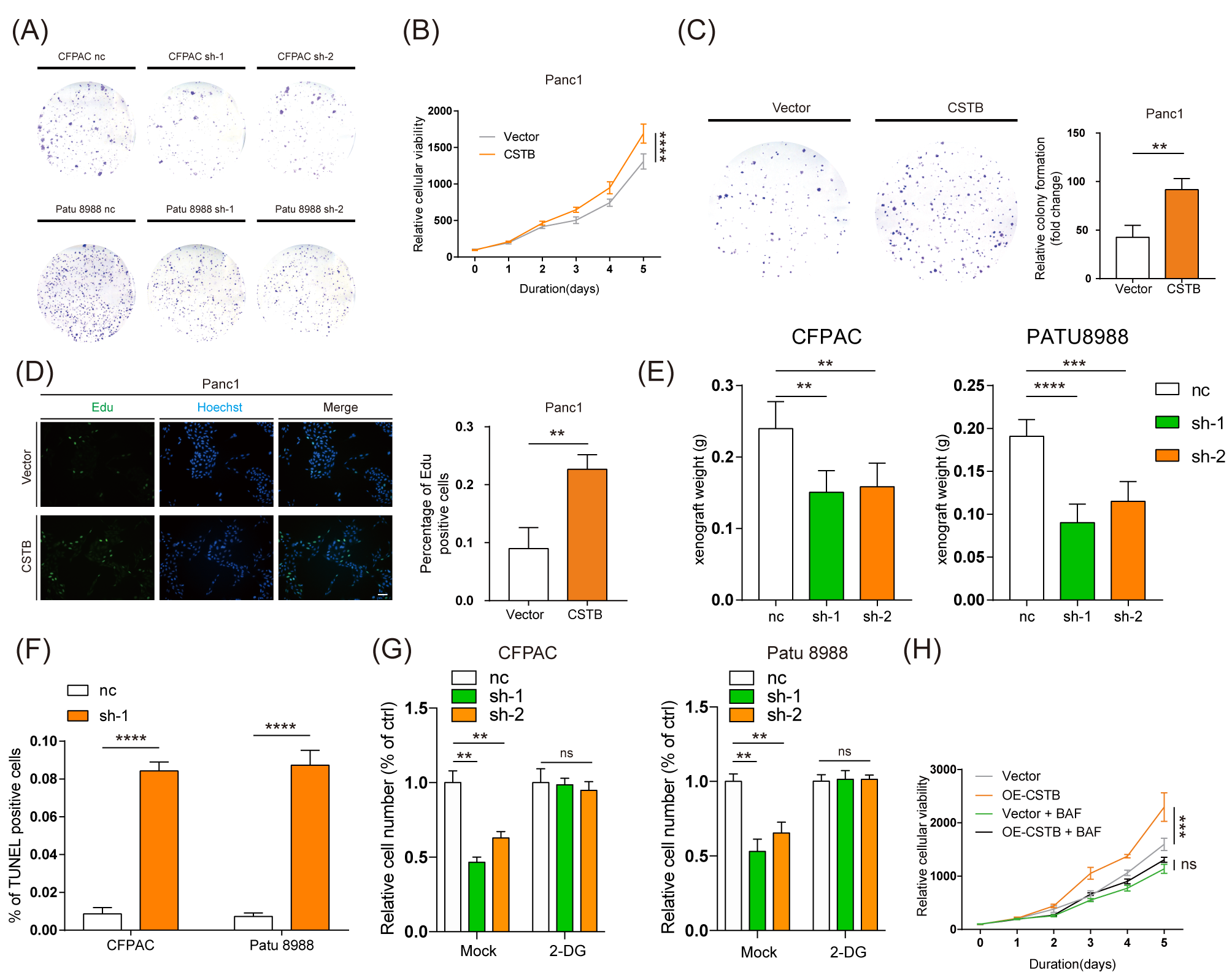

Supplement: Supplementary file 4 — Supporting Information [file CTM2-12-e1126-s010.tif]

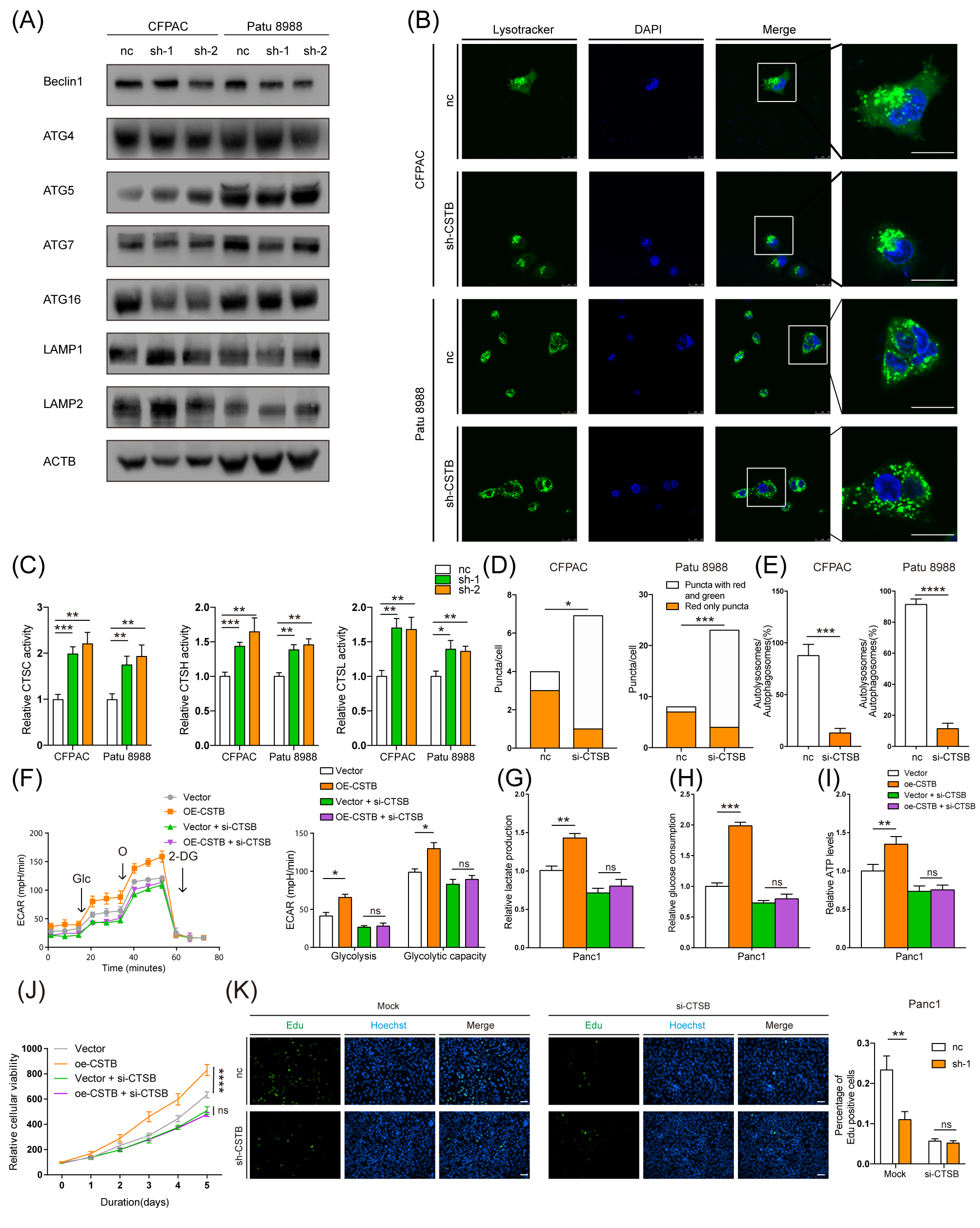

Supplement: Supplementary file 5 — Supporting Information [file CTM2-12-e1126-s015.tif]

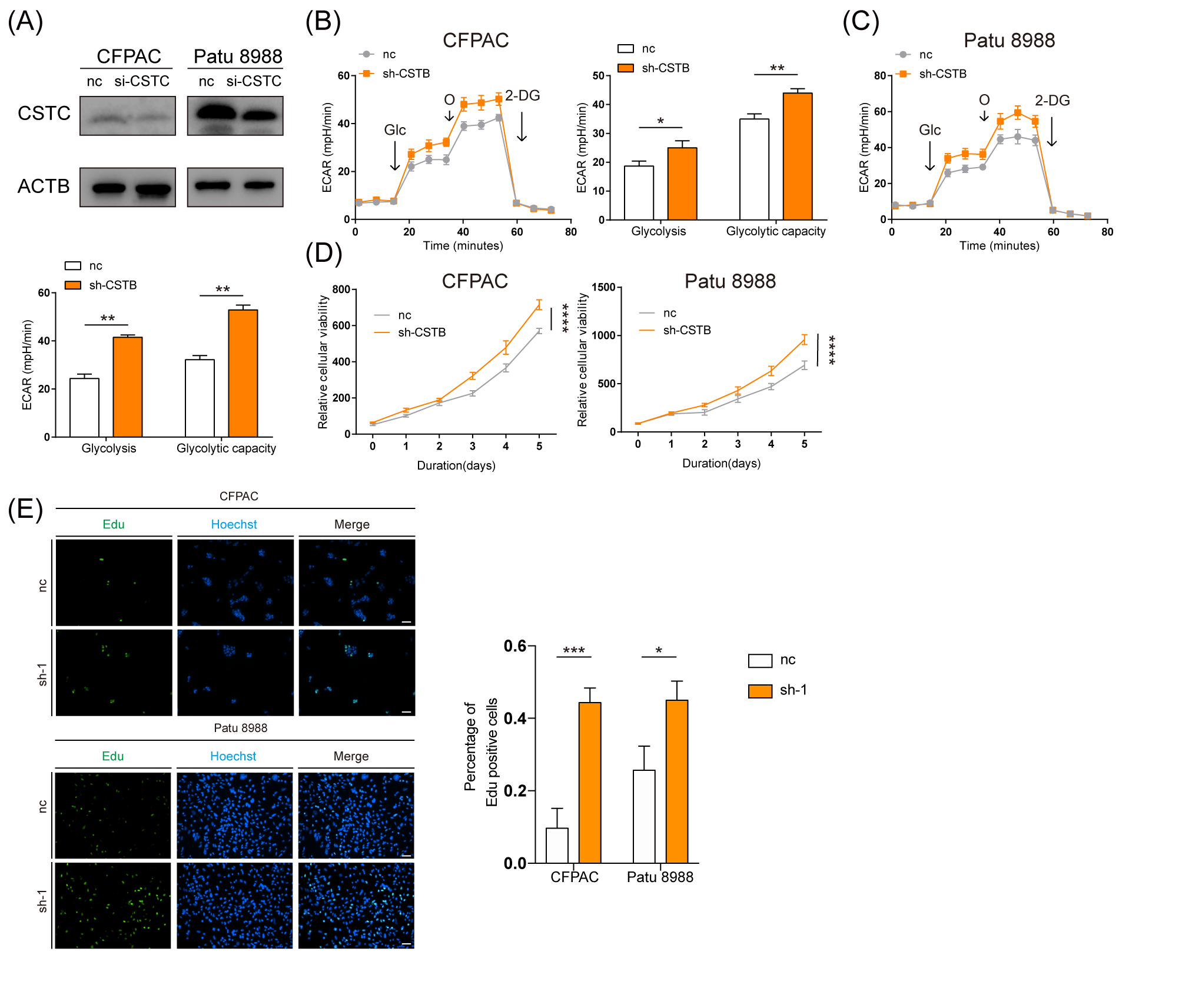

Supplement: Supplementary file 6 — Supporting Information [file CTM2-12-e1126-s006.tif]

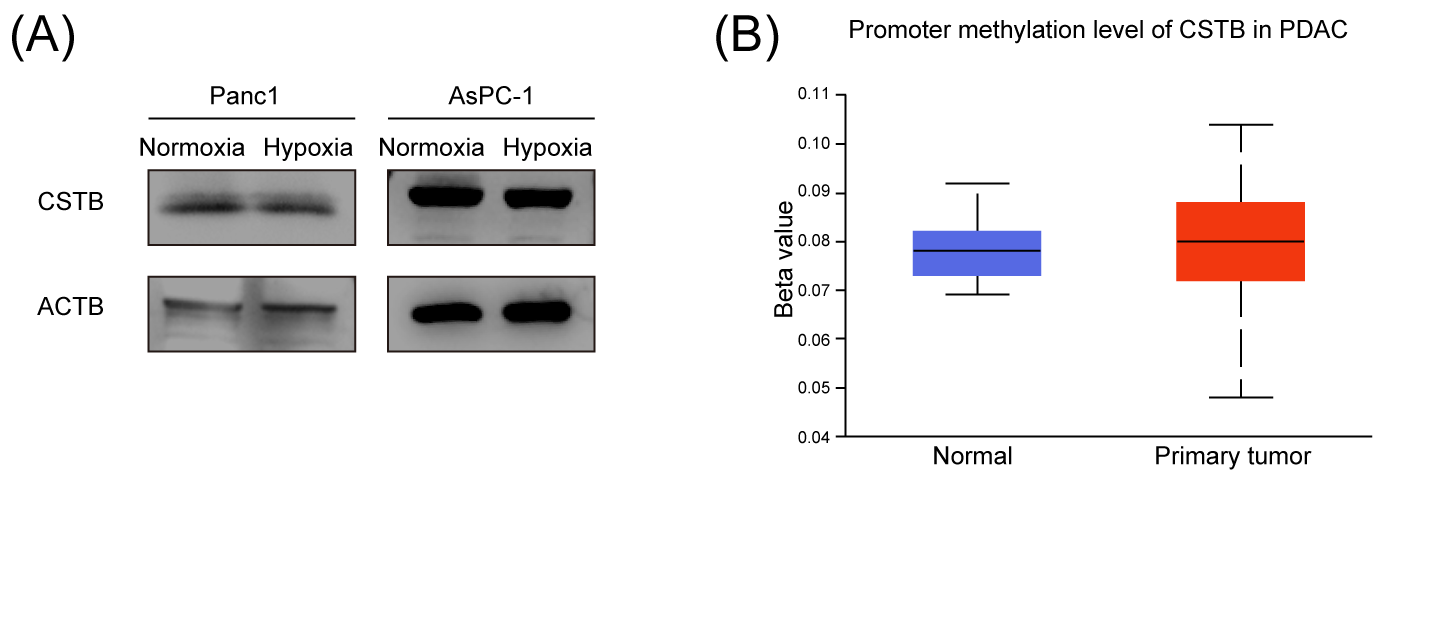

Supplement: Supplementary file 7 — Supporting Information [file CTM2-12-e1126-s008.tif]

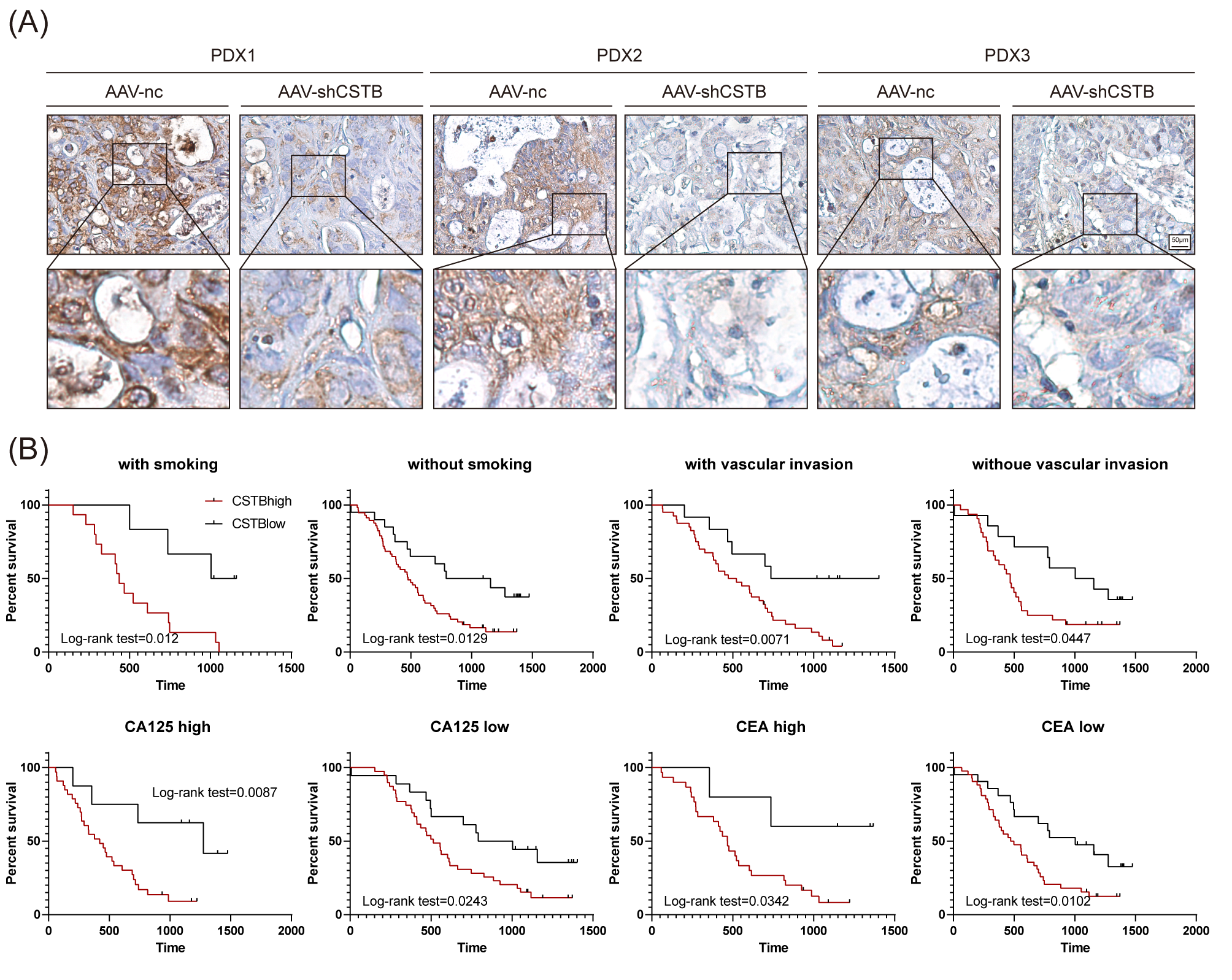

Supplement: Supplementary file 8 — Supporting Information [file CTM2-12-e1126-s005.tif]
