# Supplementary material for: Cystatin B increases autophagic flux by sustaining proteolytic activity of cathepsin B and fuels glycolysis in pancreatic cancer: CSTB orchestrates autophagy and glycolysis in PDAC
Source: Clin Transl Med. 2022 Dec 10;12(12):e1126. doi: 10.1002/ctm2.1126 (PMC9736795; doi:10.1002/ctm2.1126)
Supplement: Supplementary file 9 — Supporting Information [file CTM2-12-e1126-s007.docx]

Table S1. Autophagy signature & Glycolysis signature

| **Autophagy signature** |
| --- |
| AMBRA1；ATG5；ATG7；BAD；BAG3；BNIP3L；CAMKK2；CDC37；CLEC16A；DAPK1；DCN；DHRSX；GNAI3；GPSM1；GSK3A；GSK3B；HIF1A；HK2；HMGB1；HMOX1；IKBKG；KAT5；LACRT；LARP1；LRSAM1；MAPK3；MTDH；MUL1；NOD1；NPRL2；OPTN；PARK7；PHB2；PIK3CB；PIP4K2C；PLEKHF1；PLK2；PLK3；PRKAA1；PRKAA2；RAB12；RALB；RIPK2；RNF152；SCOC；SH3BP4；SH3GLB1；SMURF1；SPTLC1；SPTLC2+J10STK11；SUPT5H；SVIP；TFEB；TICAM1；TMEM59；TRIM14；TRIM21；TRIM22；TRIM27；TRIM38；TRIM5；TRIM6；TRIM65；TRIM8；TRIML1；TRIML2；TSC2 |
| **Glycolysis signature** |
| HK2；PDK1；PGK2；LDHA；SLC2A1；GP1；PFKL |
