# Supplementary material for: Cystatin B increases autophagic flux by sustaining proteolytic activity of cathepsin B and fuels glycolysis in pancreatic cancer: CSTB orchestrates autophagy and glycolysis in PDAC
Source: Clin Transl Med. 2022 Dec 10;12(12):e1126. doi: 10.1002/ctm2.1126 (PMC9736795; doi:10.1002/ctm2.1126)
Supplement: Supplementary file 10 — Supporting Information [file CTM2-12-e1126-s001.docx]

Table S2. Correlation Between CSTB Expression and Clinicopathologic Factors

| Factors | | Expression of CSTB | | P value |
| --- | --- | --- | --- | --- |
|  |  | Low  (n=26) | High  (n=73) |  |
| Age(y) | <65 | 19(31.1%) | 42(68.9%) | 0.240 |
|  | ≥65 | 7(18.4%) | 31(81.6%) |  |
| Sex | Male | 15(26.3%) | 42(73.7%) | 1.000 |
|  | Female | 11(26.8%) | 30(73.2%) |  |
| Tumor size | | | | 0.362 |
| <3cm | | 16(30.8%) | 36(69.2%) |  |
| ≥3cm | | 10(21.3%) | 37(78.7%) |  |
| Tumor differentiation | | | | **0.025** |
| Well/Moderate | | 22(35.5%) | 40(64.5%) |  |
| Poor | | 4(11.1%) | 32(88.9%) |  |
| N stage | | | | 0.649 |
| N0 | | 14(29.2%) | 34(70.8%) |  |
| N1/N2 | | 12(23.5%) | 39(76.5%) |  |
| M stage | | | | 0.336 |
| M0 | | 26(28.0%) | 67(72%) |  |
| M1 | | 0(0.0%) | 6(100.0%) |  |
| TNM stage | | | | 0.127 |
| I/II | | 22(31.4%) | 48(68.6%) |  |
| III/IV | | 4(14.8%) | 23(85.2%) |  |
| CA125 | | | | 0.249 |
| <23U/ml | | 18(31.0%) | 40(69.0%) |  |
| >23U/ml | | 8(19.5%) | 33(80.5%) |  |
| CA199 | | | | 0.785 |
| <25U/ml | | 6(28.6%) | 15(71.4%) |  |
| >25U/ml | | 20(25.6%) | 58(74.4%) |  |
| CEA | | | | 0.057 |
| <5ng/ml | | 21(31.8%) | 43(67.2%) |  |
| >5ng/ml | | 5(14.3%) | 30(85.7%) |  |
| Smoking | | | | 0.787 |
| Absent | | 20(26.0%) | 57(74.0%) |  |
| Present | | 6(28.6%) | 15(71.4%) |  |
| Alcohol | | | | 1.000 |
| Absent | | 21(26.6%) | 58(73.4%) |  |
| Present | | 5(27.8%) | 13(72.2%) |  |
| CSTC expression | | | | 0.496 |
| Low | | 12(30.0%) | 28(70.0%) |  |
| High | | 14(23.7%) | 45(76.3%) |  |
| SP1 expression | | | | **0.006** |
| Low | | 20(38.5%) | 32(61.5%) |  |
| High | | 6(12.8%) | 41(87.2%) |  |
