# Supplementary material for: Cystatin B increases autophagic flux by sustaining proteolytic activity of cathepsin B and fuels glycolysis in pancreatic cancer: CSTB orchestrates autophagy and glycolysis in PDAC
Source: Clin Transl Med. 2022 Dec 10;12(12):e1126. doi: 10.1002/ctm2.1126 (PMC9736795; doi:10.1002/ctm2.1126)
Supplement: Supplementary file 11 — Supporting Information [file CTM2-12-e1126-s004.docx]

Table S3. Univariate and Multivariate Cox regression analysis of potential prognostic factors in pancreatic cancer.

| Factors | Univariate | | Multivariate | |
| --- | --- | --- | --- | --- |
|  | HR (95% CI) | P value | HR (95% CI) | P value |
| Age | 1.709  (1.075-2.715) | **0.023** | 1.982  (1.200-3.273) | **0.007** |
| Gender | 1.211  (0.765-1.916) | 0.413 |  |  |
| Tumor size | 1.387  (0.880-2.187) | 0.159 |  |  |
| Tumor differentiation | 2.025  (1.265-3.241) | **0.003** | 1.433  (0.853-2.406) | 0.174 |
| N stage | 1.712  (1.083-2.707) | **0.021** | 1.666  (1.013-2.740) | **0.044** |
| M stage | 1.211  (0.488-3.004) | 0.679 |  |  |
| TNM stage | 1.417  (0.872-2.301) | 0.159 |  |  |
| Chemotherapy | 0.548  (0.348-0.862) | **0.009** | 0.477  (0.281-3.952) | **0.006** |
| Vessel invasion | 1.201  (0.759-1.901) | 0.433 |  |  |
| Smoking | 1.079  (0.634-1.835) | 0.779 |  |  |
| Alcohol | 1.402  (0.816-2.409) | 0.222 |  |  |
| CA125 | 1.283  (0.813-2.025) | 0.284 |  |  |
| CA199 | 1.704  (0.934-3.107) | 0.082 |  |  |
| CEA | 1.196  (0.750-1.909) | 0.451 |  |  |
| AFP | 0.395  (0.055-2.848) | 0.357 |  |  |
| CA724 | 0.947  (0.435-2.063) | 0.890 |  |  |
| CSTB expression | 2.640  (1.484-4.696) | **0.001** | 2.112  (1.128-3.952) | **0.019** |
| CSTC expression | 1.515  (0.944-2.432) | 0.085 |  |  |
| SP1 expression | 2.322  (1.466-3.677) | **0.0003** | 1.982  (1.200-3.273) | **0.008** |
