# Supplementary material for: Cystatin B increases autophagic flux by sustaining proteolytic activity of cathepsin B and fuels glycolysis in pancreatic cancer: CSTB orchestrates autophagy and glycolysis in PDAC
Source: Clin Transl Med. 2022 Dec 10;12(12):e1126. doi: 10.1002/ctm2.1126 (PMC9736795; doi:10.1002/ctm2.1126)
Supplement: Supplementary file 12 — Supporting Information [file CTM2-12-e1126-s014.docx]

# Extended information

| REAGENT | SOURCE | IDENTIFIER |
| --- | --- | --- |
| Antibodies |  |  |
| HK2 | Abclonal | Lot: A0994 |
| PKM | CST | Lot: 3186S |
| P-AKT | CST | Lot: 4060T |
| AKT | CST | Lot: 9272S |
| LDHA | Abclonal | Lot: A0861 |
| GAPDH | Proteintech | Lot: 60004-1-lg |
| ACTB | Proteintech | Lot: 20536-1-AP |
| SQSTM1/P62 | CST | Lot: 23214S |
| LC3A/B | CST | Lot: 12741T |
| CSTB | Proteintech | Lot: 66812-1-lg |
| Flag | CST | Lot: 14793S |
| CSTC | Proteintech | Lot: 12245-1-AP |
| Beclin1 | CST | Lot: 3738S |
| ATG4B | CST | Lot: 13507S |
| ATG5 | CST | Lot: 2630S |
| ATG7 | CST | Lot: 2631T |
| ATG16 | CST | Lot: 8689T |
| LAMP1 | CST | Lot: 9091T |
| LAMP2 | Abcam | Lot: ab199946 |
| CTSB | Proteintech | Lot: 12216-1-AP |
| CTSC | Abclonal | Lot: A8403 |
| CTSL | Proteintech | Lot: 10938-1-AP |
| CTSH | Proteintech | Lot: 10935-1-AP |
| H3K27ac | CST | Lot: 8173T |
| Goat-anti-rabit | Proteintech | Lot: 20000311 |
| Anti-Mouse-IgG | Proteintech | Lot: 20000261 |
| 488-anti-Mouse | Servicebio | Lot: GB25301 |
| Cys3-anti-Rabblit | Servicebio | GB21303 |
| Plasmids |  |  |
| sh1-CSTB | Genechem | CCGGCAGACCAACAAAGCCAAGCA  CTCGAGATGCTTGGCTTTGTTGGTC  TGTTTTTG |
| sh2-CSTB | Genechem | CCGGCTGTGTTTAAGGCCGTGTCAT  CTCGAGATGACACGGCCTTAAACAC  AGTTTTTG |
| OE-CSTB | Genechem | Seeing below |
| mut-CSTB | Genechem | Seeing below |
| sh-CSTC | Genechem | CCGGCGTGGCTGGAGTGAACTATTT  CTCGAGAAATAGTTCACTCCAGCCA  CGTTTTTG |
| pGL3-promoter | Bioegene |  |
| CSTB-promoter | Bioegene | Seeing below |
| Mut-CSTB-promoter | Bioegene | Seeing below |
| siRNA |  |  |
| CTSB | Bioegene | GGUUUCUGGUGGCCUCUAUGATT |
| CSTC | Bioegene | AAATAGTTCACTCCAGCCACG |
| SP1 | Bioegene | CCAACAGAUUAUCACAAAUTT |
| TFAP2A | Bioegene | GGGUAUUAACAUCCCAGAUTT |
| Primers |  |  |
| GLUT1-F  GLUT1-R | Sangon | ATTGGCTCCGGTATCGTCAA  GCTCAGATAGGACATCCAGGGTA |
| LDHA-F  LDHA-R | Sangon | ATGGCAACTCTAAAGGATCAGC  CCAACCCCAACAACTGTAATCT |
| HK2-F  HK2-R | Sangon | AGCCCTTTCTCCATCTCCTT  GCTTGCCTACTTCTTCACGG |
| ENO1-F  ENO1-R | Sangon | GCCGTGAACGAGAAGTCCTG  ACGCCTGAAGAGACTCGGT |
| PKM-F  PKM-R | Sangon | ATGTCGAAGCCCCATAGTGAA  TGGGTGGTGAATCAATGTCCA |
| TPI1-F  TPI1-R | Sangon | AGCTCATCGGCACTCTGAAC  CCACAGCAATCTTGGGATCT |
| CSTB-F  CSTB-R | Sangon | AGGTCCCAGCTTGAAGAGAAA  CGCAGGTGTACGAAGTCCTC |
| Primer#1-F  Primer#1-R | Sangon | CCGAGACCCAGCACATCG  CAAAGCGGCTTCTTTCGCTC |
| Primer#2-F  Primer#2-R | Sangon | GGGACTCCGAAGCCAAAGTG  GAATCTGGCGAGGGGACTC |
| Primer#3-F  Primer#3-R | Sangon | CAACCCACCGGCGACA  GCTGGTATCGTCTTTCCGGG |
| Primer#4-F  Primer#4-R | Sangon | CAGCCTTCAGTGTTCTCGGT  CAGTCGGAGTAGGCGCAAT |
| Primer#5-F  Primer#5-R | Sangon | ATTCCACCAGAGAACCCTGC  CAGGGAGGGGGAGACGG |

Sequence for CSTB(NM_000100) plasmid

ATGATGTGCGGGGCGCCCTCCGCCACGCAGCCGGCCACCGCCGAGACCCAGCACATCGCCGACCAGGTGAGGTCCCAGCTTGAAGAGAAAGAAAACAAGAAGTTCCCTGTGTTTAAGGCCGTGTCATTCAAGAGCCAGGTGGTCGCGGGGACAAACTACTTCATCAAGGTGCACGTCGGCGACGAGGACTTCGTACACCTGCGAGTGTTCCAATCTCTCCCTCATGAAAACAAGCCCTTGACCTTATCTAACTACCAGACCAACAAAGCCAAGCATGATGAGCTGACCTATTTC

Sequence for mutant CSTB(C.149G>A)

ATGATGTGCGGGGCGCCCTCCGCCACGCAGCCGGCCACCGCCGAGACCCAGCACATCGCCGACCAGGTGAGGTCCCAGCTTGAAGAGAAAGAAAACAAGAAGTTCCCTGTGTTTAAGGCCGTGTCATTCAAGAGCCAGGTGGTCGCGGAGACAAACTACTTCATCAAGGTGCACGTCGGCGACGAGGACTTCGTACACCTGCGAGTGTTCCAATCTCTCCCTCATGAAAACAAGCCCTTGACCTTATCTAACTACCAGACCAACAAAGCCAAGCATGATGAGCTGACCTATTTC

Cstb-promoter(-350)

TGCGCGGCCCGGACCCCGCCACCCTGCAGGATTGCGCCTACTCCGACTGCCCCTTCCCTATCGTCCCACCCTGCGCGCCCAACCCACCGGCGACACCCGGCCGCGCCCCCGCCCCGGTCCGTGTGACTCGGCGCCCGGAAAGACGATACCAGCCCCGGGAGGGGGGCGCTCCCTCCCGACACCAGCGCTGGGCGCGGAGACCCAGCCTGCGGCGAGTGGTGGCCAGGTTCCCCGCCCCGCGCCCCGCCCCGCGCCCCGCCCCGCGCGTCCCTTCTTGCGGGGCCACCGCGACCCCGCAGGGGACTCCGAAGCCAAAGTGCCTCCTCCCCGCCCCTTGGTTCCGCCCGCGCGTCACGTGACCCCAGCGCCTACTTGGGCTGAGGAGCCGCCGCGTCCCCTC

Cstb-promoter(-350)-mut

TGCGCGGCCCGGACCCCGCCACCCTGCAGGATTGCGCCTACTCCGACTGCCCCTTCCCTATCGTCCCACCCTGCGCGCCCAACCCACCGGCGACACCCGGCCGCGAAAAACAAAGAGTCCGTGTGACTCGGCGCCCGGAAAGACGATACCAGCCCCGGGAGGGGGGCGCTCCCTCCCGACACCAGCGCTGGGCGCGGAGACCCAGCCTGCGGCGAGTGGTGGCCAGGTTCCCCGCCCCGCGCCCCGCCCCGCGCCCCGCCCCGCGCGTCCCTTCTTGCGGGGCCACCGCGACCCCGCAGGGGACTCCGAAGCCAAAGTGCCTCCTCCCCGCCCCTTGGTTCCGCCCGCGCGTCACGTGACCCCAGCGCCTACTTGGGCTGAGGAGCCGCCGCGTCCCCTC
