# Supplementary material for: Cystatin B increases autophagic flux by sustaining proteolytic activity of cathepsin B and fuels glycolysis in pancreatic cancer: CSTB orchestrates autophagy and glycolysis in PDAC
Source: Clin Transl Med. 2022 Dec 10;12(12):e1126. doi: 10.1002/ctm2.1126 (PMC9736795; doi:10.1002/ctm2.1126)
Supplement: Supplementary file 16 — Supporting Information [file CTM2-12-e1126-s012.pptx]

## Slide 1
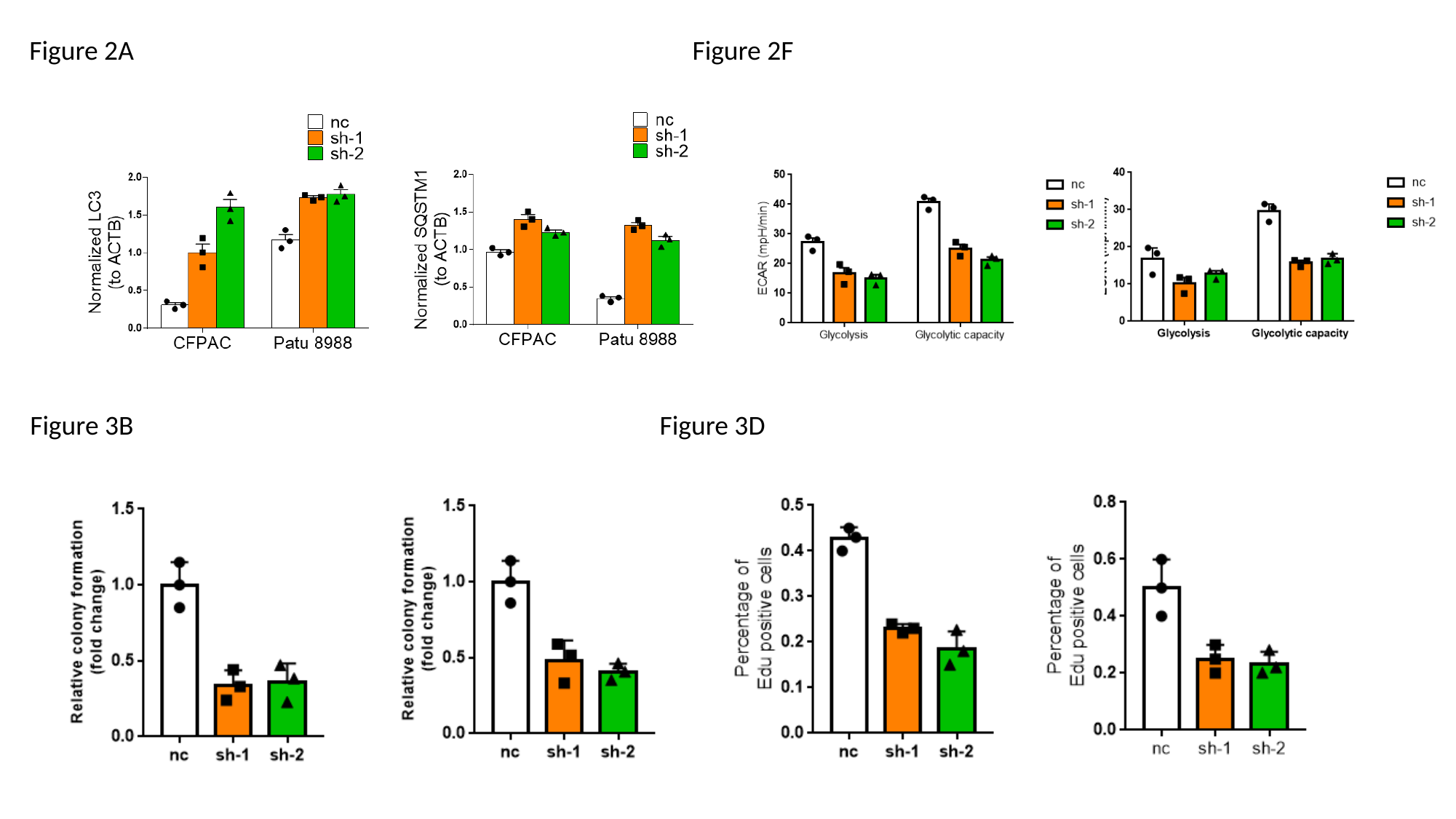

Figure 2F
Figure 2A
Figure 3D
Figure 3B

## Slide 2
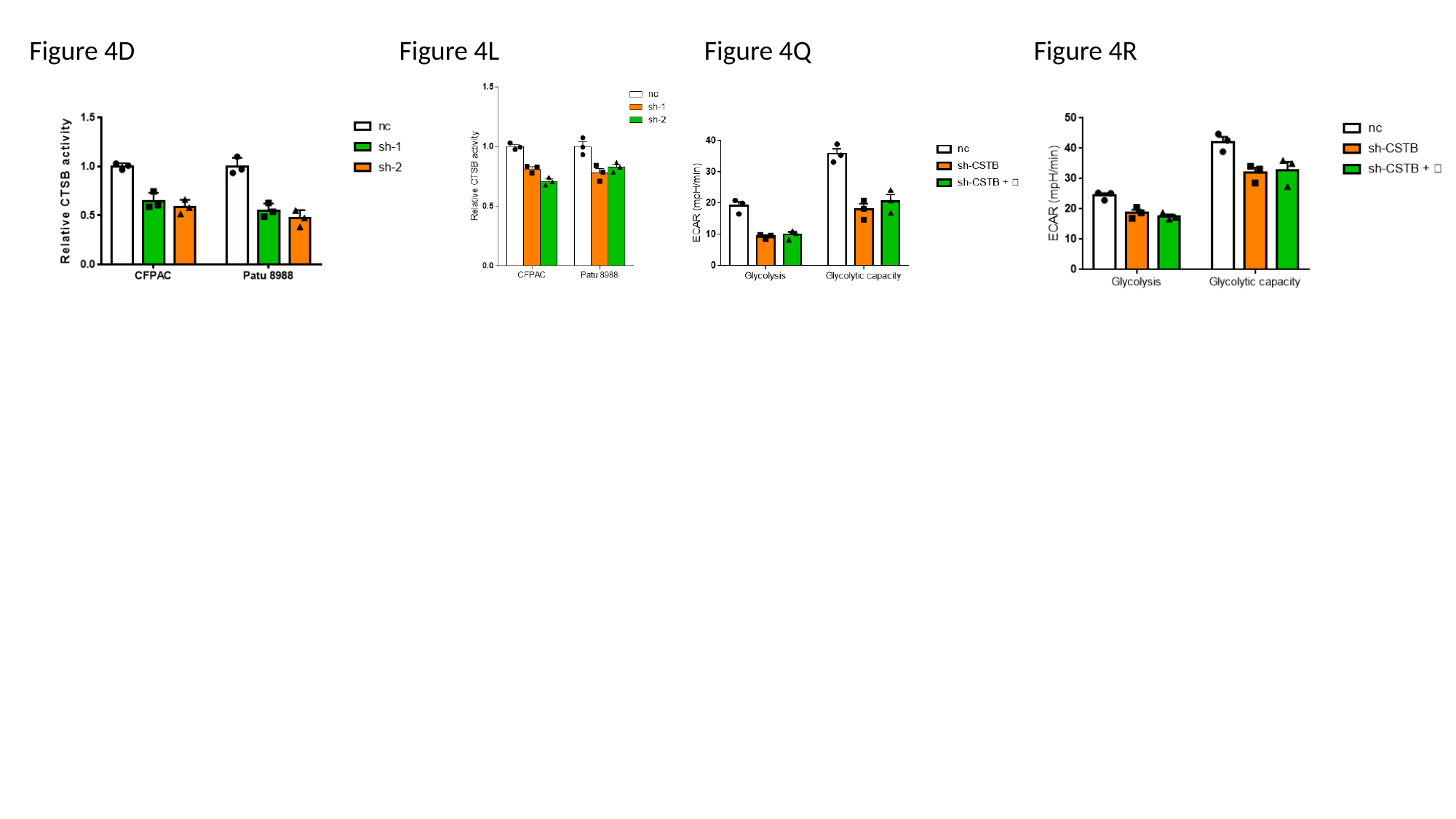

Figure 4D
Figure 4L
Figure 4Q
Figure 4R

## Slide 3
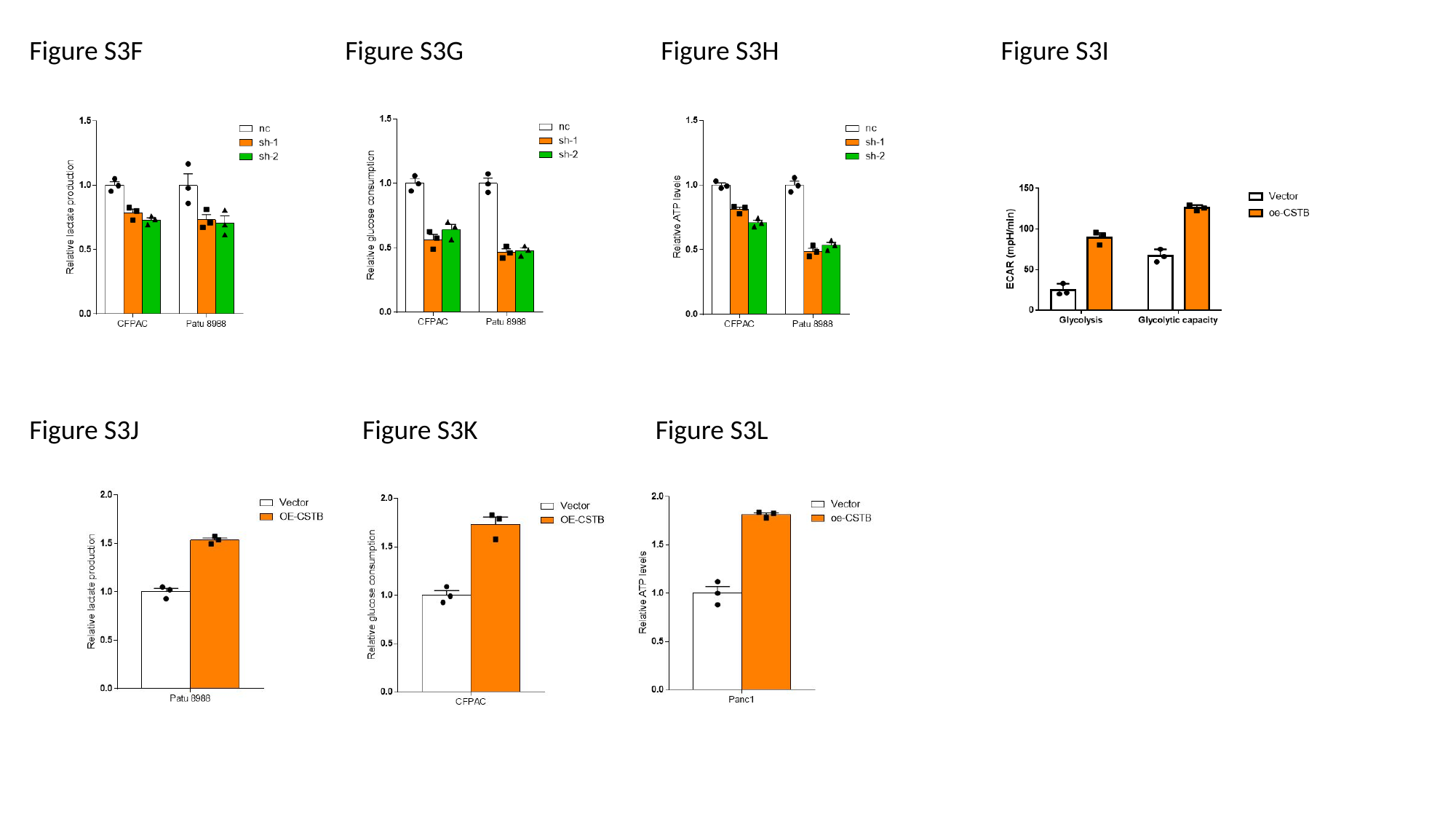

Figure S3F
Figure S3G
Figure S3H
Figure S3I
Figure S3K
Figure S3L
Figure S3J

## Slide 4
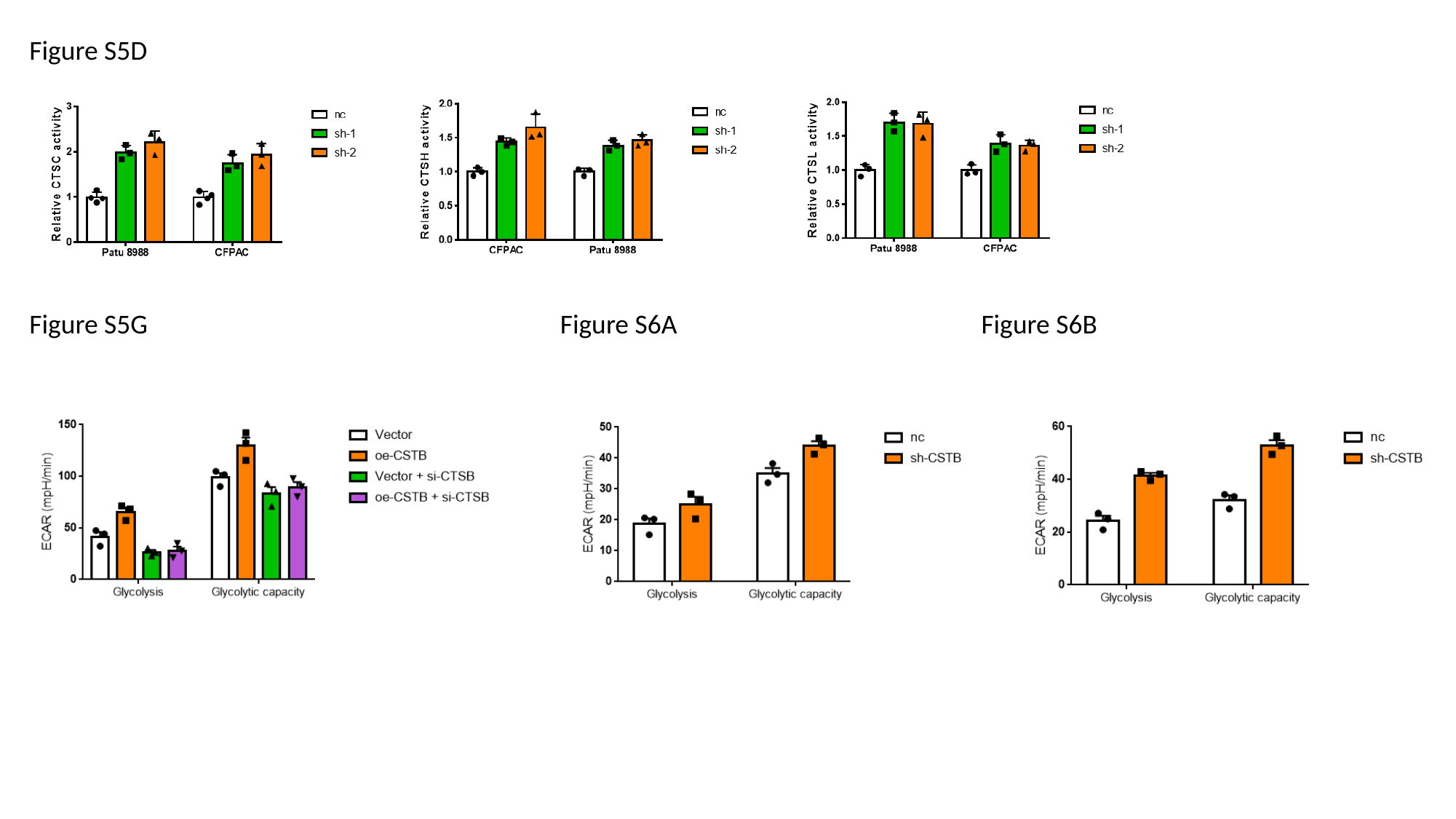

Figure S5D
Figure S5G
Figure S6A
Figure S6B
